# Re-replication of a Centromere Induces Chromosomal Instability and Aneuploidy

**DOI:** 10.1371/journal.pgen.1005039

**Published:** 2015-04-22

**Authors:** Stacey L. Hanlon, Joachim J. Li

**Affiliations:** 1 Department of Biochemistry and Biophysics, University of California, San Francisco, San Francisco, California, United States of America; 2 Department of Microbiology and Immunology, University of California, San Francisco, San Francisco, California, United States of America; Duke University, UNITED STATES

## Abstract

The faithful inheritance of chromosomes during cell division requires their precise replication and segregation. Numerous mechanisms ensure that each of these fundamental cell cycle events is performed with a high degree of fidelity. The fidelity of chromosomal replication is maintained in part by re-replication controls that ensure there are no more than two copies of every genomic segment to distribute to the two daughter cells. This control is enforced by inhibiting replication initiation proteins from reinitiating replication origins within a single cell cycle. Here we show in *Saccharomyces cerevisiae* that re-replication control is important for the fidelity of chromosome segregation. In particular, we demonstrate that transient re-replication of centromeric DNA due to disruption of re-replication control greatly induces aneuploidy of the re-replicated chromosome. Some of this aneuploidy arises from missegregation of both sister chromatids to one daughter cell. Aneuploidy can also arise from the generation of an extra sister chromatid via homologous recombination, suggesting that centromeric re-replication can trigger breakage and repair events that expand chromosome number without causing chromosomal rearrangements. Thus, we have identified a potential new non-mitotic source of aneuploidy that can arise from a defect in re-replication control. Given the emerging connections between the deregulation of replication initiation proteins and oncogenesis, this finding may be relevant to the aneuploidy that is prevalent in cancer.

## Author Summary

The stable inheritance of genetic information requires an elaborate mitotic machinery that acts on the centromeres of chromosomes to ensure their precise segregation. Errors in this segregation can lead to aneuploidy, an unbalanced chromosomal state in which some chromosomes have different copy number than others. Because aneuploidy is associated with developmental abnormalities and diseases such as cancer, there is considerable interest in understanding how these segregation errors arise. Much of this interest has focused on identifying defects in proteins that make up the mitotic machinery. Here, we show that defects in a completely separate process, the control of DNA replication initiation, can lead to chromosome segregation errors as a result of inappropriate re-replication of centromeres. Similar deregulation of replication initiation proteins has been observed in primary human tumors and shown to promote oncogenesis in mouse models. Together, these results raise the possibility that centromeric re-replication may be an additional source of aneuploidy in cancer. In combination with our previous work showing that re-replication is a potent inducer of gene amplification, these results also highlight the versatility of re-replication as a source of genomic instability.

## Introduction

During their life cycle, cells must duplicate their genome exactly once, then precisely segregate the two copies into their daughter cells. In eukaryotes, elaborate regulatory controls ensure that each of these processes occur with great fidelity. Because DNA replication and chromosome segregation are such distinct processes occurring at opposite stages of the cell cycle, these controls are usually studied independently of each other.

The initiation of DNA replication is regulated at thousands of replication origins scattered throughout eukaryotic genomes [1–3]. Origins are licensed in G1 phase for later initiation in S phase by the loading of the core replicative helicase Mcm2–7 by the origin recognition complex (ORC), Cdc6, and Cdt1. This licensing is restricted to one round per cell cycle by multiple mechanisms that inhibit these licensing proteins after they have executed their function. Thus, after origins initiate and the replicative helicases move away with the replication forks, they cannot relicense or reinitiate for the remainder of the cell cycle. Much of this block to relicensing is mediated by cyclin-dependent kinases (CDKs) through phosphorylation and/or direct binding. In addition, in metazoans Cdt1 is inhibited by replication-coupled proteolysis and binding to inhibitory proteins called geminins. Each of these many mechanisms contribute to minimizing the probability of reinitiation, as deregulation of these mechanisms leads to progressively more reinitiation as more mechanisms are compromised [4,5]. Moreover, preserving such high fidelity of reinitiation control is critical for genomic stability as reinitiation and re-replication is an extremely potent source of segmental amplifications and duplications [6].

After chromosomal replication, faithful segregation of the resulting sister chromatids requires the correct bipolar attachment of sister centromeres to microtubules emanating from opposite poles of the mitotic spindle [7,8]. This bi-orientation of sister chromatids is established in mitosis at kinetochore complexes, which are assembled onto centromeres and serve as attachment sites for microtubules. For proper bi-orientation each sister chromatid must be attached to microtubules from only one pole. Rings of cohesin complexes are thought to embrace both sister chromatids with greatest density around their centromeres, preventing their premature separation and allowing the detection of tension across sisters when they become bi-oriented [9]. The absence of this tension is sensed by the spindle assembly checkpoint, which prevents anaphase until all sister chromatid pairs become bi-oriented [10]. When anaphase proceeds, the cohesin rings are cleaved, releasing each chromatid to be pulled to the spindle pole to which they are attached.

Importantly, the faithful segregation of sister chromatids depends on proper assembly of kinetochores, correct establishment of centromeric cohesion, and the presence of only one centromere per sister chromatid. In principle, each of these factors can be disrupted by re-replication through a centromere, raising the possibility that the fidelity of reinitiation control is important for the fidelity of chromosome segregation.

To test the dependence of segregation fidelity on reinitiation control, we asked whether transient and localized re-replication of a centromere could disrupt the segregation of a chromosome in the budding yeast *Saccharomyces cerevisiae*. We find that centromeric re-replication is a potent way of inducing missegregation of both sister chromatids to one daughter cell. Surprisingly, we also discover that centromeric re-replication can induce aneuploidy by formation of an extra sister chromatid. This formation is dependent on homologous recombination, suggesting that centromeric re-replication can lead to chromosomal breaks that then undergo homologous recombination to reconstitute intact chromatids. Finally, microscopic examination of re-replicated centromeres suggests that they have the ability to reassemble functional kinetochores and be placed under tension. In summary, the deregulation of DNA replication initiation can have a significant impact on the mitotic mechanisms that ensure faithful chromosome segregation and can provide a potential new source of chromosomal instability and aneuploidy. These findings have potential relevance to cancer where both compromised reinitiation control and defective segregation fidelity can be found.

## Results

We previously demonstrated that the cell-cycle controls preventing reinitiation of replication are critical for genome stability by showing that compromising these controls leads to intrachromosomal amplifications [6]. In those studies we developed a system in which conditional deregulation of replication initiation proteins can induce transient and localized re-replication of any chromosome segment of interest. This system provided an opportunity to explore how the regulation of DNA replication influences the execution of mitosis by allowing us to induce the re-replication of a centromere.

To examine if re-replication could affect chromosomal stability, we designed an assay to quantify the segregation fidelity of a budding yeast chromosome following the transient, localized re-replication of its centromere. We had previously shown that conditional deregulation of a specific subset of DNA replication controls makes origins susceptible to reinitiation, with the most prominent and detectable reinitiation occurring at the origin *ARS317* [4,6]. To induce overt re-replication of a centromere, we inserted a cassette containing *ARS317* 8 kb from the Chromosome V centromere (*CEN5*) on one of the two homologs in a diploid re-replicating strain. To monitor the copy number of the re-replicating chromosome, the cassette also carried the copy-number reporter *ade3–2p*, which makes cells containing zero, one, or more copies white, pink, or red, respectively [11].

**Fig 1:**
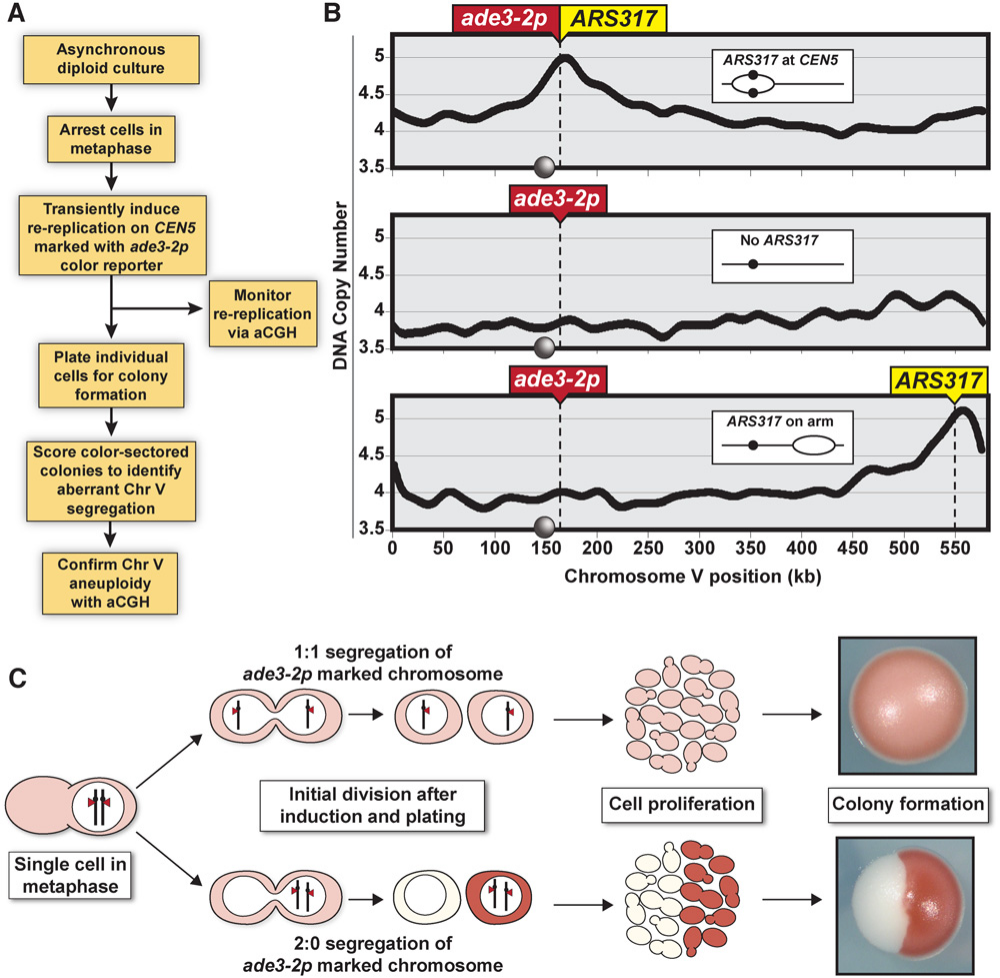
Monitoring chromosome segregation fidelity after centromeric re-replication. (A) Experimental flowchart starting with diploid re-replicating cells containing one Chromosome V homolog marked with the *ade3–2p* copy number reporter. (B) Re-replication profile of Chromosome V for diploid cells arrested in metaphase (with baseline copy number of 4C) and induced to re-replicate (see S1 Table). *ARS317* and *ade3–2p* mark integration sites of the preferentially reinitiating origin and the copy number reporter, respectively. Inset shows schematic of re-replication bubbles inferred from profiles. Circles on X-axis and in schematic represent centromere *CEN5*. (C) Illustration showing how 1:1 segregation of the *ade3–2p* marked homolog in the first cell division after centromere re-replication leads to pink colonies and 2:0 missegregation leads to red/white sectored colonies.

In our assay (Fig. 1A), exponentially growing cells were allowed to proceed through a normal S phase before being arrested in metaphase using the spindle inhibitor nocodazole. At the arrest, re-replication was transiently induced until half of the *ARS317* in the population had reinitiated, as measured by array comparative genomic hybridization (aCGH) (Fig. 1B). Cells were released from the arrest either before or after the induction of re-replication by plating for individual colonies. These colonies were screened for colored sectors that suggested the *ade3–2p* marked Chromosome V homolog missegregated in the mitosis following the induced re-replication. Normal segregation of one sister chromatid to each daughter cell (1:1 segregation) produces a uniformly pink colony. However, missegregation of both sister chromatids to one daughter cell (2:0 segregation) would leave that cell with both copies of the *ade3–2p* reporter and the other daughter with none, generating colonies divided into large red and white sectors (Fig. 1C).

**Fig 2:**
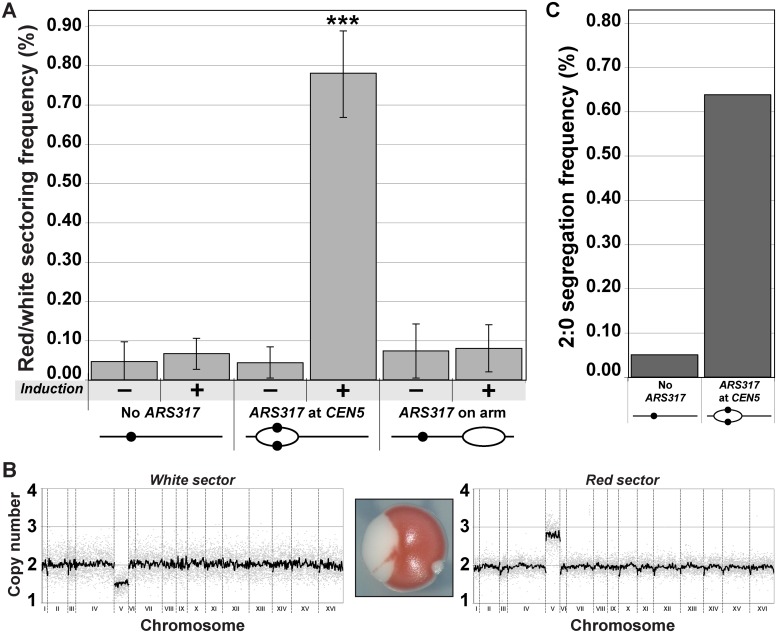
Centromeric re-replication causes 2:0 missegregation of chromosomes. (A) Centromeric re-replication induces red/white sectored colonies. Diploid re-replicating strains (characterized in Fig. 1B and induced to re-replicate as described in Fig. 1A) were scored for the frequency of red/white sectored colonies either before (-) or after (+) induction of re-replication (see S7 Table). Frequencies are presented as the mean ± SD (n ≥ 3). When compared to no *ARS317* at *CEN5*, the frequency after re-replication was significantly different for *ARS317* at *CEN5* (***, *p* = 6.03x10^–8^) but not for *ARS317* on the right arm (*p* = 0.175). (B) aCGH copy number analysis of a representative red/white colony that was scored as a 2:0 segregation event (see Materials and Methods). (C) Estimated frequency of 2:0 segregation events after 3 hr of re-replication. The average sectoring frequency for each strain shown in (A) was multiplied by the fraction of aCGH-analyzed isolates that showed 2:0 segregation of the *ade2–3p* marked Chromosome V homolog (see S2 and S8 Tables).

The frequency of red/white sectored colonies observed after centromeric re-replication was 7.8 x 10^–3^, nearly 20 times the frequency observed in colonies plated before the induction of re-replication (Fig. 2A). In contrast, this induction did not significantly stimulate the frequency of red/white colonies in a congenic strain lacking *ARS317* or a strain where *ARS317* was relocated near the right end of Chromosome V (Fig. 2A). In the latter strain, reinitiation from *ARS317* was too far away to cause measurable *CEN5* re-replication (Fig. 1B, bottom panel), demonstrating that it was specifically centromeric re-replication that induced the high frequency of red/white colony formation.

To determine if the red/white sectored colonies induced by *CEN5* re-replication were genotypically consistent with a 2:0 missegregation of the re-replicated Chromosome V homolog, we used aCGH to assess genomic copy number in each sector. Assuming that the unmarked non-re-replicating homolog segregates normally 1:1, we would expect the red sector to contain a total of three copies of Chromosome V and the white sector to contain one copy. Nine out of 11 red/white sectored colonies showed such a distribution of Chromosome V (Fig. 2B and S2 Table), suggesting that a large proportion of the red/white sectored colonies scored likely arose from a 2:0 missegregation event. Of the much fewer red/white colonies derived from the re-replicating strain completely lacking *ARS317*, a similar proportion (3/4) also corresponded to colonies that had undergone 2:0 missegregation of the *ade3–2p* marked Chromosome V homolog (S2 Table).

This aCGH analysis allowed us to estimate the frequency of apparent 2:0 missegregation events from the frequency of red/white colonies (Fig. 2C, S8 Table). Centromeric re-replication induced by *ARS317* caused a missegregation frequency of 6.4 x 10^–3^ in the following mitosis, approximately 12-fold higher than the missegregation frequency observed in the absence of *ARS317*. We suspect that the latter frequency is itself elevated both because of the prolonged nocodazole arrest [12,13] and because of cryptic re-replication occurring throughout the genome independent of *ARS317* (and possibly involving *CEN5*) [4,14]. Hence, we were most interested in comparing the re-replication-induced frequency of Chromosome V 2:0 missegregation events to the spontaneous frequency of these events. Although the latter has not been directly measured for Chromosome V in wild-type diploid cells, upper limits can be estimated by the spontaneous rate of Chromosome V loss (2–8 x 10^–6^ per cell division [15,16]) and gain (3 x 10^–7^ per cell division [17]), respectively. Thus the frequency of 2:0 missegregation events induced by transient centromeric re-replication in a single cell cycle is approximately 10^3^–10^4^ higher than the expected spontaneous frequency of these events. We conclude that centromeric re-replication can be a potent inducer of chromosomal instability and aneuploidy.

**Fig 3:**
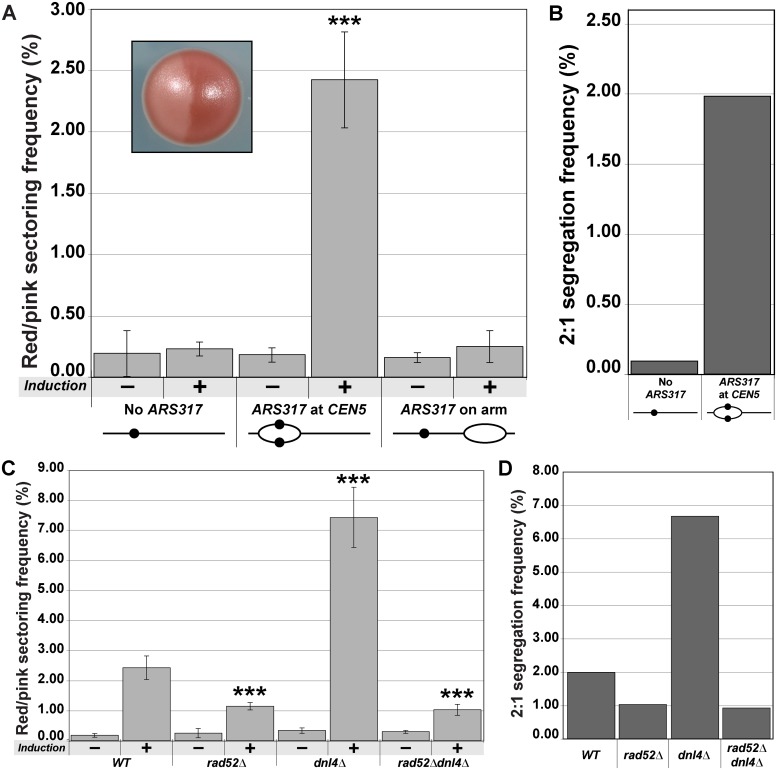
Centromeric re-replication causes 2:1 segregation through chromosome gain. (A) Centromeric re-replication induces red/pink sectored colonies. Diploid re-replicating strains characterized in Fig. 1B and induced to re-replicate as described in Fig. 1A, were scored for the frequency of red/pink sectored colonies (see inset) either before (-) or after (+) induction of re-replication (see S7 Table). Frequencies are presented as the mean ± SD (n ≥ 3). When compared to no *ARS317* at *CEN5*, the frequency after re-replication was significantly different for *ARS317* at *CEN5* (***, *p* = 3.3x10^–8^) but not for *ARS317* on the right arm (*p* = 0.253). (B) Estimated frequency of 2:1 segregation events after 3 hr of re-replication. The average sectoring frequency for each strain shown in (A) was multiplied by the fraction of aCGH-analyzed isolates that showed 2:1 segregation of the *ade2–3p* marked Chromosome V homolog (see S3 and S8 Tables). (C) Dependence of red/pink colony frequencies induced by centromeric re-replication on recombination. Diploid re-replicating strains with reinitiating origin *ARS317* at *CEN5* and homozygous deletions of the indicated genes were scored for the frequency of red/pink sectored colonies as described and presented as in (A) (see S7 Table). When compared to the undeleted WT background, the frequency after re-replication was significantly different for *rad52∆* (***, *p* = 5.8x10^–6^), *dnl4∆* (***, *p* = 2.1x10^–9^), and *rad52∆ dnl4∆* (***, *p* = 7.4x10^–5^). (D) Dependence of 2:1 segregation events induced by centromeric re-replication on homologous recombination. Segregation events were estimated as described in (B) using the frequencies reported in (C) (see S3 and S8 Tables).

Surprisingly, we also discovered a second source of aneuploidy induced by centromeric re-replication from *ARS317*. Colonies divided into large red and pink sectors were observed following centromeric re-replication at a frequency of 2.4 x 10^–2^, ten-fold higher than the frequency of red/pink colonies observed in a strain lacking *ARS317* (Fig. 3A). The color of the sectors suggested the presence of one copy of the *ade3–2p* marked Chromosome V homolog in the pink sector and at least two copies in the red sector, consistent with there being three copies of the re-replicating homolog segregating in a 2:1 manner. For most of these red/pink colonies, this 2:1 segregation was confirmed by aCGH data showing a total of three copies of Chromosome V in the red sector and two copies in the pink sector (S3 Table). Using the aCGH data to convert red/pink colony frequencies to 2:1 segregation frequencies (S8 Table), we observed that strains with *ARS317* driving centromeric re-replication induced these events at a frequency of 2.0 x 10^–2^, 22-fold higher than strains without *ARS317* (Fig. 3B). This induction of 2:1 segregation suggests that centromeric re-replication can induce chromosome gain by forming a whole additional copy of the chromosome. Upper limits on the spontaneous frequency of these 2:1 events can be estimated by the spontaneous rate of chromosome gain, 3 x 10^–7^ per cell division [17]. Thus the frequency of 2:1 missegregation events induced by transient centromeric re-replication in a single cell cycle is approximately 10^4^ to 10^5^ higher than the expected spontaneous frequency of these events.

A trivial explanation for chromosome gain is that, despite the predominantly localized nature of the re-replication induced by *ARS317* on Chromosome V (Fig. 1B), a small fraction of these chromosomes somehow manage to re-replicate to completion. If that were the case, however, we would expect the red-pink colony frequency to be independent of the chromosomal location of *ARS317*. Using the strain with *ARS317* relocated near the right end of Chromosome V to minimize centromeric re-replication (Fig. 1B), we found that the red/pink colony frequency was similar to that of the strain completely lacking *ARS317* (Fig. 3A). We thus conclude that the extra copy of Chromosome V detected in the red/pink colonies is dependent on centromeric re-replication, suggesting that they do not arise from re-replication of the entire chromosome.

An alternative route for generating these extra chromosomes is suggested by our previous observation that re-replication forks are highly susceptible to breakage and subsequent recombination [18]. Hence, we wondered whether the extra chromosomes in our red/pink colonies could have arisen from double-strand break repair. Eukaryotes rely primarily on two pathways to repair double-strand breaks [19–21]. One is homologous recombination, which in budding yeast is dependent of Rad52, a protein that can facilitate complementary strand annealing and single-strand exchange [22,23]. The other is nonhomologous end joining, which depends on Dnl4, the budding yeast ortholog of DNA Ligase IV [24]. To test if double-strand break repair plays a role in the induction of 2:1 segregation events, we performed centromeric re-replication experiments in *rad52∆*, *dnl4∆*, and *rad52∆ dnl4∆* backgrounds.

Deletion of *RAD52* reduced the induced frequency of red/pink colonies (Fig. 3C) and 2:1 segregation events (as determined by aCGH) (Fig. 3D; S3 and S8 Tables) by approximately half. Thus a significant proportion of 2:1 segregation events induced by centromeric re-replication depend on homologous recombination and are unlikely to be due to complete re-replication of Chromosome V. Curiously, the induced frequency of red/pink colonies and 2:1 segregation events nearly tripled when *DNL4* was deleted (Fig. 3C and 3D; S3 and S8 Tables). Moreover, the vast majority of these additional colonies and segregation events were dependent on the presence of *ARS317* (S1 Fig), as well as the presence of *RAD52*, as demonstrated by the drop to *rad52∆* levels in the *rad52∆ dnl4∆* double deletion strain (Fig. 3C and 3D). Importantly, none of the changes in frequencies observed with any of the deletions could be attributed to changes in re-replication efficiency because the re-replication profiles of the deletion mutants were comparable to that of the wild-type strain (S2 Fig). In addition, the effect of the deletions was specific to the 2:1 segregation events because the red-white colony frequencies associated with 2:0 missegregation events were not affected by the deletions (S3 Fig). Together these results suggest that DNA damage induced by centromeric re-replication can be efficiently repaired by homologous recombination in a manner that generates an extra whole sister chromatid. This route to aneuploidy appears to be partially inhibited by nonhomologous end joining, possibly by competition for the damage substrate [19,20].

**Fig 4:**
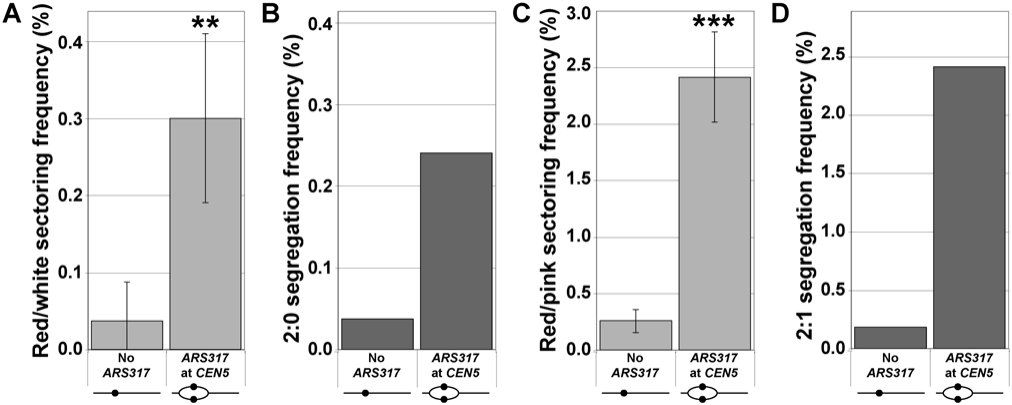
Centromeric re-replication induced in cycling cells causes 2:0 and 2:1 segregation. Re-replication was induced for 3 hr in unarrested cycling cells (see S4 Fig), which were then analyzed as described in Fig. 1A. (A) Frequencies of red/white sectored colonies after re-replication (see S7 Table) are presented as the mean ± SD (n ≥ 3) and shown to be significantly higher for *ARS317* at *CEN5* versus no *ARS317* at *CEN5* (** *p* = 0.002). (B) Estimates of 2:0 segregation frequencies after re-replication were calculated as described for Fig. 2C (see S4 and S8 Tables). (C) Frequencies of red/pink sectored colonies after re-replication (see S7 Table) are presented as the mean ± SD (n ≥ 3) and shown to be significantly higher for *ARS317* at *CEN5* versus no *ARS317* at *CEN5* (*** *p* = 2.2x10^–5^). (D) Estimates of 2:1 segregation frequencies after re-replication were calculated as described for Fig. 3B (see S5 and S8 Tables).

By temporally isolating the centromeric re-replication of a chromosome from its normal replication and segregation, the experimental strategy used above provided the cleanest demonstration that re-replication induces 2:0 and 2:1 segregation events. This strategy, however, left open the possibility that the spindle disruption and/or metaphase arrest caused by nocodazole was also required for the induction of these events [12,13]. Hence, we asked if centromeric re-replication induced in unarrested, asynchronously-dividing cells was sufficient to induce 2:0 and 2:1 events. We activated re-replication for three hours in these cells, which induced equivalent amounts of centromeric re-replication as that induced by three hours of re-replication in nocodazole-arrested cells (S4 Fig). The state of the cells plated after the re-replication was also comparable in that they had undergone re-replication for approximately one cell cycle before the DNA damage caused by re-replication [25–28] triggered their transient arrest in mitosis (S5 Fig). In the absence of nocodazole, we still observed strong induction of red/white and red/pink colonies relative to a strain lacking *ARS317* (Fig. 4A and 4C). Analysis of chromosomal copy number using aCGH confirmed that this colony induction reflected increases in the frequencies of 2:0 and 2:1 segregation events (Fig. 4B and 4D; S4, S5, and S8 Tables). Thus centromeric re-replication induced in cycling cells is sufficient to generate these events.

As a first step toward exploring the molecular events that lead from centromeric re-replication to chromosome missegregation or breakage we examined the mitotic behavior of re-replicated centromeres by fluorescence microscopy. The re-replicating *CEN5* was fluorescently marked with *tet* operator arrays placed 2 kb to the left of the centromere in haploid cells expressing tdTomato-tagged Tet repressors. At this distance, bipolar spindle tension placed on normal bi-oriented sister centromeres in metaphase can be detected by the separation of sister arrays into two resolvable fluorescent spots [29–31]. To monitor the position of the marked centromeres relative to the mitotic spindle, the microtubule subunit Tub1 was tagged with GFP. To examine the interaction of the spindle with re-replicated centromeres in metaphase, we took advantage of the fact that cycling cells induced to re-replicate trigger a DNA damage response that causes them to arrest in metaphase (S5 Fig, [26]). At this point, we can observe the opposing action of bipolar spindle tension and pericentromeric cohesion on centromeres.

**Fig 5:**
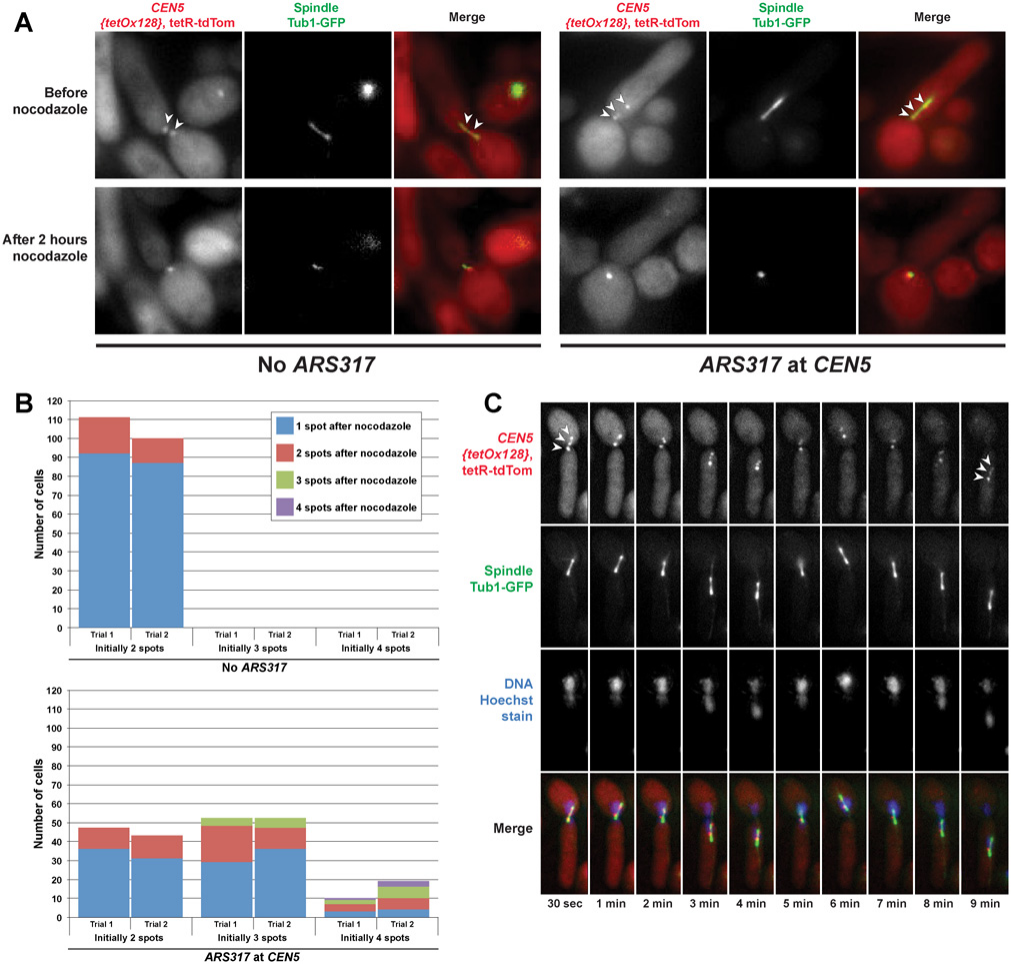
Spindle dependent dynamic separation and movement of re-replicated centromeres. Exponentially-growing cells with either *ARS317* integrated near *CEN5* (YJL10671/YJL10672) or not integrated at all (YJL10665/YJL10666) were induced to re-replicate for three hours by addition of galactose. During this time, the DNA damage response triggered by re-replication caused both strains to arrest in metaphase with intact mitotic spindles. Cells were shifted to dextrose containing media to limit further induction of re-replication before being imaged live, initially in the absence of nocodazole then 2 hr after addition of nocodazole. (A) Spots corresponding to the *TET* operator arrays positioned near *CEN5* and bound to tdTomato-tagged *TET* repressors are indicated by arrowheads. (B) Quantification of the number of spots observed before and after nocodazole addition in cells with *ARS317* near *CEN5* or without *ARS317*. The number of cells scored pre-nocodazole is charted based on initial spot number, with each bar divided into the number of cells retaining one, two, three, or four spots after nocodazole treatment (see S6 Table). Each strain in both trials was scored for ≥ 100 cells. (C) Video microscopy in a single Z-plane of a live cell that has undergone centromeric re-replication. The three spots corresponding to *TET* operator arrays bound to tdTomato-tagged *TET* repressors (on the left of *CEN5*) are indicated by arrowheads in the first and last panels.

When *ARS317* was not present to reinitiate replication near *CEN5*, all metaphase-arrested cells displayed two resolvable fluorescent spots during periodic imaging over a 20 min period (Fig. 5A, left panel). This was consistent with the “centromere breathing” expected of bi-oriented sister centromeres [30,32]. In contrast, when *ARS317* was positioned near *CEN5* so that ~40–50% of these centromeres re-replicated, cells displaying three or four spots were observed (Fig. 5A, right panel, and 5B). The total number of additional spots observed was 33–40% above that expected for bi-oriented sister centromeres in the absence of centromeric re-replication. This implies that a large proportion of the re-replicated centromeres of a sister chromatid can be pulled apart from each other as well as from the centromere (or possibly re-replicated centromeres) of the other sister chromatid. During the periodic imaging, both the non-re-replicated and re-replicated centromeres remained mostly separated while the spindle and centromeres were pulled in various directions around the nucleus (S6 Fig). The dynamic nature of these movements can be seen with higher resolution time-lapse imaging of re-replicated cells containing three spots (Fig. 5C; S1 and S2 Movie). These results are consistent with many of the re-replicated centromeres undergoing separation due to bipolar spindle tension.

To determine if the separation of re-replicated centromeres was indeed dependent on spindle tension, the cells we scored for spot numbers were continuously imaged after treatment with nocodazole, which inhibits microtubule polymerization. For the cells without *ARS317* near *CEN5*, approximately 85% of the sister centromere pairs collapsed to a single spot as the mitotic spindle disappeared, similar to a previously published quantification of the effects of nocodazole on centromere breathing [33]. This collapse is due to pericentromeric cohesion, which resists the spindle tension placed on bi-oriented sister centromeres. For cells with *ARS317* near *CEN5*, most of those with two or three spots ended up with one spot displaying little directed motion (Fig. 5A and 5B). This collapse of three spots to one indicates that many of these re-replicated centromeres were separated because of spindle tension. It further implies that these re-replicated centromeres reassembled functional kinetochores, maintained pericentromeric cohesion, and underwent bipolar spindle attachments.

We note that, although many cells with four resolvable spots showed a reduction in the number and motion of spots upon nocodazole addition, most did not collapse down to a single spot. There were also a few cells with three spots that retained all three after nocodazole treatment. These observations suggests that in some cases pericentromeric cohesion of re-replicated centromeres may be compromised, particularly if more than one centromere is re-replicated.

## Discussion

### A novel connection between the fidelity of re-replication control and the fidelity of chromosome segregation

We have shown that centromeric re-replication provides a highly potent way to induce chromosomal instability and aneuploidy in cells where the mitotic segregation machinery is intrinsically intact. This suggests that the mitotic mechanisms that preserve segregation fidelity are not designed to handle the problems that arise when centromeres are re-replicated. As a consequence chromosome segregation fidelity is dependent on the fidelity of re-replication control, establishing an important connection between these very distinct processes. Moreover, as discussed later, it raises the possibility that the decreased fidelity of both processes that is seen in cancer cells may be related.

### Centromeric re-replication induces missegregation

The aneuploidy arising from centromeric re-replication is generated in part through missegregation of both sister chromatids to one daughter cell. Exactly how this missegregation occurs remains to be determined, but centromeric re-replication has the potential to perturb the segregation machinery in at least three ways: (1) disruption of kinetochores; (2) disruption of centromeric cohesion; and (3) attachment of a single sister chromatid to microtubules from both spindle poles (i.e., bipolar attachment).

In budding yeast, kinetochores inherited from the previous cell cycle are disrupted by passage of the replication fork but then rapidly reassemble onto the newly replicated centromeres [34]. In principle, a similar disruption followed by reassembly could occur with re-replication forks since budding yeast kinetochores can assemble and become functional throughout the cell cycle [35]. Moreover, our observation that more than two centromeres in a re-replicating strain can be microscopically resolved in a microtubule dependent manner is consistent with the presence of functional kinetochores on many re-replicated centromeres. Nonetheless, we cannot rule out some of the missegregation we detected in our sectored colony assay arising from a failure to reassemble kinetochores on a minority of re-replicated centromeres.

The microtubule dependence of the separation of re-replicated centromeres also suggests that pericentromeric cohesion is often preserved following centromeric re-replication. Such cohesion is presumably responsible for the collapse of re-replicated and separated *CEN5* spots to a single spot following the disruption of microtubules. We note, however, that in the less frequent cases when four spots were present in a cell, they often collapsed to two spots rather than one, suggesting that in some instances, particularly when more than one centromere re-replicates, pericentromeric cohesion may be compromised.

**Fig 6:**
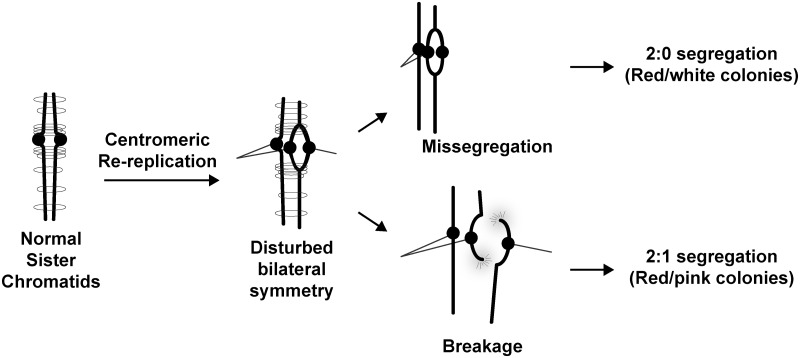
Possible ways for centromeric re-replication to perturb chromosome segregation. Normal sister chromatids are bilaterally symmetric and held together via cohesin to ensure their bi-orientation with respect to the spindle poles. Centromere re-replication disrupts this bilateral symmetry and can lead to abnormal bipolar attachment of a single chromatid to both spindle poles. During anaphase, this bipolar attachment could lead to a 2:0 segregation pattern. Alternatively the affected sister chromatid could break and repair in a *RAD52-*dependent manner to produce a 2:1 segregation pattern. Also conceivable but not shown are disruption of kinetochore function or pericentromeric cohesion by re-replication.

For the majority of centromeric re-replication bubbles that retain functional kinetochores and pericentromeric cohesion, attachment of re-replicated centromeres to microtubules from opposite poles will result in bipolar attachment of the re-replicated sister chromatid to the mitotic spindle (Fig. 6). Our observation of three or four resolvable centromeres moving around the nucleus in a microtubule-dependent manner is consistent with such bipolar attachments.

Importantly, this potential source of missegregation can, in principle, be established without triggering the two surveillance mechanisms that normally ensure faithful segregation. One of these mechanisms prevents merotelic kinetochore attachments, i.e. bipolar attachment of microtubules to individual kinetochores. Normally, because each sister chromatid has only a single kinetochore, this mechanism plays a critical role in preventing bipolar attachment of the chromatid to the mitotic spindle. However, if centromeres re-replicate, the presence of two kinetochores on a sister chromatid permits its bipolar attachment to the spindle without requiring merotelic attachment to either kinetochore. The second mechanism, the spindle assembly checkpoint, which detects the absence of tension on kinetochores, would also not be triggered because the tension generated by bipolar attachment to re-replicated kinetochores would be transmitted to their sister kinetochore(s) via sister chromatid cohesion. The inability of either surveillance mechanisms to sense and correct bipolar attachment to centromerically re-replicated sister chromatids could help explain why centromeric re-replication is such a potent inducer of missegregation and aneuploidy.

It should be noted that the re-replication bubbles that enable these bipolar attachments are transient chromosomal structure. Exactly how these bubbles eventually disappear is not clear, although they can conceivably be resolved during the normal course of a subsequent S phase. Nonetheless, because the bubbles are transient, centromeric re-replication can induce simple aneuploidy in a hit-and-run fashion. This stands in contrast to dicentric chromosomes, whose bipolar attachments inevitably lead to chromosome breakage and rearrangement [36–38].

### Centromeric re-replication induces chromosome gain

In addition to inducing aneuploidy by missegregating sister chromatids, centromeric re-replication can also induce aneuploidy by the formation of extra sister chromatids, as manifested by the appearance of 2:1 segregation events. The dependence of at least half of these events on *RAD52*, a gene essential for most homologous recombination in budding yeast [39], implies that many of these extra chromatids are generated by chromosome breakage and recombinational repair.

The breakage is not surprising. It may arise from bipolar spindle tension being placed on centromeric re-replication bubbles by, or it might arise simply because of the susceptibility of re-replication forks to breakage [18]. What is striking, is the apparent efficiency with which the repair of these breaks can be channeled into the formation of extra sister chromatids. Again, this outcome contrasts sharply with the chromosomal rearrangements that result from dicentric chromosome breaks [37], and it reinforces the notion that chromosomes with re-replicated centromeres are not simply dicentric chromosomes in a different guise. Whether similar generation of aneuploidy by extra sister chromatid formation can occur in mammalian cells with their much larger chromosomes remains to be seen. Nonetheless, our studies have uncovered a novel way in which aneuploidy can be generated.

It is possible that centromeric re-replication can also lead to other chromosomal consequences that we did not observe either because they are lethal or because we did not score them. For example, the large rise in *RAD52*-dependent 2:1 segregation events in a *dnl4* mutant background suggests that there may be other competing fates for chromosome breakage events that involve nonhomologous end-joining. In our primary colony screen, we focused on colonies that were significantly induced by centromeric re-replication and that we anticipated would be most straightforward to interpret, namely red-white and red-pink sectored colonies. Analysis of other colonies with different or more complex shapes and color patterns may uncover other types of chromosomal loss, gain, or rearrangements induced by centromeric re-replication.

### A potential new source of aneuploidy in cancer

The connection we have established between decreased fidelity of re-replication control and decreased fidelity of chromosome segregation may be relevant to cancer as compromised fidelity for both processes have been observed in cancer cells. The decreased fidelity of chromosome segregation is well established in cancer [40]. Approximately 90% of solid tumors and 50% of hematopoietic cancers are aneuploid, with many exhibiting chromosomal instability [41,42]. Moreover, there are increasing hints that aneuploidy may contribute to tumorigenesis by promoting genomic instability [43,44].

How chromosomal instability arises in cancers is still an open question. Increasing attention has been placed on non-mitotic perturbations that can disrupt chromosome segregation because mitotic genes directly involved in kinetochore function, spindle function, cohesion, or the spindle assembly checkpoint are rarely mutated in sequenced cancer genomes [40,45]. One such perturbation is the accumulation of excess centrosomes, the microtubule organizing centers at the poles of spindles, as this can lead to incorrect merotelic attachment of sister chromatids to more than one spindle pole [46]. However, despite being present in many cancers, excess centrosomes are not observed in all cancers displaying chromosomal instability [47], raising the question of what other non-mitotic perturbations contribute to this instability.

Our observation that centromeric re-replication is a potent inducer of aneuploidy offers one such perturbation. This possibility is encouraged by accumulating evidence that re-replication may occur in cancer and contribute to oncogenesis [3,48,49]. Specifically, moderately elevated levels of the replication initiation proteins Cdc6 and Cdt1, which in high amounts induces detectable re-replication in cell culture and model organisms [27,50–55], has been observed in multiple types of primary tumors [56–61]. In addition, moderate overexpression of Cdt1 has been shown to potentiate carcinogenesis in mouse models [58,62,63]. Re-replication has yet to be directly confirmed in any of these settings [50,58,62,63], but this is likely due to the fact that levels of re-replication currently detectable by conventional replication assays cause extensive DNA damage and cell lethality [5,25–28,50,51,64–66]. Hence, only lower levels of re-replication can be compatible with cancer cell viability, and detecting such cryptic re-replication will require the development of more sensitive replication assays [67].

Finally, re-replication that does occur in human cells may well involve centromeric DNA, as this DNA was found to be preferentially re-replicated when overt re-replication was induced in *Schizosaccharomyces pombe* or S2 cells of *Drosophila melanogaster* [68,69]. Indeed, cryptic centromeric re-replication could account for the chromosomal instability that was induced in the absence of detectable re-replication by moderate overexpression of Cdt1 in normal human fibroblasts [70].

In summary, cryptic re-replication of centromeres could be occurring in cancer cells with deregulated initiation proteins, and our work shows that such re-replication is highly potent at inducing chromosomal instability and aneuploidy. Further investigation will be needed to determine whether this hypothesis applies to specific cancers, but our work offers a potential source of chromosomal instability and aneuploidy that has not been considered before. When combined with our previous observation that re-replication is a potent inducer of segmental amplifications and duplications [6], this work also displays the versatility with which re-replication can induce genomic instability.

## Materials and Methods

### Oligonucleotides

Oligonucleotides used for disrupting *RAD52*, *DNL4*, or *HMRa*, inserting the *URA3* marker, or inserting the targeting marker *TRP1* for the *tet* operator (*tetO*) array insertion are listed in S11 Table.

### Plasmids

The plasmids pSH006 and pSH005 were constructed for integrating the copy number reporter *ade3–2p [11]* with or without *ARS317*, respectively, at ChrV_160kb near *CEN5*. The plasmid pSH006 contains the *kanMX-ade3–2p-ARS317* cassette excised from pBJL2890 [6] with XbaI and StuI, and pSH005 contains the *kanMX-ade3–2p* cassette excised from pBJL2889 [6] with XbaI and StuI. In both plasmids the cassettes are flanked by homology sequences from ChrV_160 as listed in S10 Table. To make pSH008, pSH006 was cut with BsaBI and XbaI, filled in, and ligated to remove a secondary NotI site.

The plasmids pSH020 and pSH019 were constructed for integrating *ade3–2w* with or without *ARS317*, respectively, at ChrV_548kb near the right end of Chromosome V. The plasmid pSH020 contains a *natMX-ade3–2w-ARS317* cassette based on the cassette in pSH008 but with *natMX* replacing *kanMX* and *ade3–2w* replacing *ade3–2p*. The allele *ade3–2w* is a derivative of *ade3–2p* where a frameshift mutation has been engineered at the beginning (between +45 and 46) of the open reading frame by inserting a single nucleotide. The cassette in pSH020 is flanked by homology sequences from ChrV_548 that are listed in S10 Table. The plasmid pSH019 is equivalent to pSH005 but has no *ARS317* in the cassette.

The plasmid pSH013 was constructed to introduce *bar1∆* by two-step gene replacement. It contains genomic sequences upstream and downstream of the *BAR1* open reading frame (separated by a SpeI restriction site) cloned into pRS306 [71].

The plasmid pSR14 was obtained from the Dave Morgan Lab (UCSF) with permission from its original source, the Susan Gasser Lab (Friedrich Miescher Institute). This plasmid contains 128 tandem copies of *tetO*, the *LEU2* marker, and target sequence for its integration into the genome [72].

The plasmid pCUP1-TetR-tdTomato-ADE2 was obtained from Dan Liu in the Dave Morgan Lab (UCSF). It expressed a Tet repressor (TetR) linked to the fluorescent protein tdTomato under the control of the *pCUP1* promoter. The fusion construct was integrated into the middle of *ADE2*, and the entire fragment inserted into pRS406. The fusion protein lacks a nuclear localization sequence.

The plasmid pBJL2667 expresses GFP-Tub1 fusion protein under the control of the *pHIS3* promoter. This plasmid contains *pHIS3-GFP-TUB1* fusion construct from the multiple cloning site of pRS306 in the plasmid pAFS91 (Aaron Straight, Stanford) [73] subcloned into multiple cloning site of pRS304.

Sequences files for all plasmids are available upon request.

### Strain construction

All haploid and diploid strains used in our experiments (S9 Table) are in a genetic background that can conditionally induce reinitiation of DNA replication, most prominently from the origin *ARS317*. They also have one homolog of Chromosome V marked with the *ade3–2p* copy number reporter to the right its centromere *CEN5* (ChrV_160kb). This *ade3–2p* marked homolog also contains *ARS317* close to the centromere *CEN5* (ChrV_160kb), *ARS317* near the right end of the chromosome (ChrV_548kb), or no *ARS317* at all. The basic strategy for generating these diploids was to mate a *MATa* haploid strain containing the *ade3–2p* marked Chromosome V with a congenic *MATα* strain containing a *URA3* marked Chromosome V.

All haploid strains used in these matings were derived from the haploid strain YL3155 [6] (*MATa ORC2-(NotI*, *SgrAI) orc6(S116A) leu2 ura3–52 trp1–289 ade2 ade3 MCM7–2NLS bar1*::*LEU2 CAN1 HMRa*). YJL3155 is primed to reinitiate DNA replication because the *MCM7–2NLS* allele makes the Mcm2–7 complex constitutively nuclear [74], and the *orc6(S116A)* partially prevents CDK inhibition of the origin recognition complex (ORC) by mutating one of the 11 CDK consensus phosphorylation sites in ORC [75]. To generate the haploid strains used in the matings, various combinations of changes to the following loci were made:

*MATα*—*MATα* haploids were obtained by switching from *MATa* using *pGAL-HO* in pSB283 [76].

*hmra∆*::*hphMX*—for both haploids, the endogenous *ARS317* was removed by deleting the entire *HMRa* locus. This was done by integrating an *hmra∆*::*hphMX* disruption fragment that was generated by two-step PCR amplification from pAG26 [77] using primers described in S11 Table.

*bar1∆*—for both haploids, *bar1*::*LEU2* was converted to *bar1∆* by loop-in/loop-out gene replacement using BsrG1 linearized pSH013.

*ura3∆*::*{ACT1term-pGAL1/10-delntCDC6*,*cdk2A-CDC6term}*—for both haploids, a galactose-inducible, stabilized version of *CDC6* was integrated in place of the *uras3–52* allele by loop-in/loop-out gene replacement with SmaI linearized pKJF019 [18].

*ChrV_160*::*{kanMX*, *ade3–2p*, *±ARS317}—*for the *MATa* haploids, *CEN5* was marked with the copy number reporter *ade3–2p* by integration of a cassette containing *kanMX* and *ade3–2p* at ChrV_160kb (right of the centromere). When *ARS317* was to be positioned near *CEN5*, we used a {*kanMX*, *ade3–2p*, *ARS317}* cassette excised from pSH008 with SacI and NotI. When *ARS317* was not to be on the chromosome or to be located on the right arm (at ChrV_548kb), we used a {*kanMX*, *ade3–2p}* cassette excised from pSH005 with SacI and NotI.

*ChrV_160*::*URA3*—for the *MATα* haploids *URA3* was integrated near *CEN5* by one step gene insertion with a *URA3* integration fragment generated by two step PCR amplification from pRS316 using the primers described in S11 Table.

*ChrV_548*::*{natMX*, *ade3–2w*, *±ARS317}—*for the *MATa* haploids, a cassette containing *natMX* and *ade3–2w* (the inactive color reporter described above) was integrated near the right end at ChrV_568kb. When *ARS317* was to be positioned at this location we used a {*natMX*, *ade3–2w*, *ARS317*} cassette excised from pSH020 excised with SacI and NotI. When *ARS317* was not to be on the chromosome or to be located near *CEN5*, we used a {*natMX*, *ade3–2w*} cassette excised from pSH019 with SacI and NotI.

*dnl4∆*—to make diploid strains homozygous for the *dnl4* deletion, *DNL4* in both *MATa* and *MATα* haploids was deleted by integrating a *dnl4∆*::*LEU2* disruption fragment that was generated by two step PCR amplification from pRS315 [71] using the primers described in S11 Table.

*rad52*∆—to make diploid strains homozygous for the *rad52* deletion but wild-type for *DNL4*, *RAD52* in both *MATa* and *MATα* haploids was deleted by integrating a *rad52∆*::*LEU2* disruption fragment that was generated by two step PCR amplification from pRS315 using the primers described in S11 Table. To make diploid stains homozygous for both *rad52* and *dnl4*, *MATa* and *MATα* haploids that already contained the *dnl4∆*::*LEU2* allele had their *RAD52* genes deleted using a different marker. A *rad52∆*::*URA3* disruption fragment that was generated by two step PCR amplification from pAG36 [77] was used for the *MATa* haploids, and a *rad52∆*::*natMX* disruption fragment that was generated by two step PCR amplification from pAG36 was used for the *MATα* haploids. Primers used for the PCR are listed in S11 Table.

*trp1–289*::*{GFP-TUB1*, *TRP1}—*the plasmid pBJL2667 was linearized with Bsu36I and integrated via loop-in at the endogenous *trp1–289* locus.

*ade2*::*{pCUP1-TetR-tdTomato*, *URA3*, *ADE2}—*the plasmid pCUP1-TetR-tdTomato-ADE2 was linearized with BglII and looped-in at the endogenous *ade2* locus. Due to the inability of a functional *ADE2* to confer complete adenine prototrophy without a fully functional *ADE3*, cells were selected for the *URA3* marker in the backbone of the plasmid. However, the presence of a fully functional *ADE2* prevents accumulation of the red pigment produced in an *ade3–2p ade2* background, meaning colonies were now without color (white).

*ChrV_151*::*{LEU2*, *tetOx128}—*The plasmid pSR14 was linearized with AscI and stability integrated into ChrV at 151 kb, which is to the left of *CEN5* (the re-replication cassette is to the right of the centromere). To integrate this construct at the proper location, targeting homology was generated and integrated first. A fragment containing *TRP1* and specific targeting sequence was generated by PCR amplification from pRS314 using primers described in S11 Table. This construct was placed at ChrV_151, which provided the targeting homology for the sequences at the end of the linearized pSR14. Cells were visually inspected for bright red spots prior to strain archiving.

### Media and cell growth

Cells were grown in or on YEP medium [78] supplemented with 2% wt/vol dextrose (YEPD), 8% wt/vol dextrose (YEP8D), or 3% wt/vol raffinose + 0.05% wt/vol dextrose (to form YEPRd). The color development plates were synthetic base with 2% wt/vol dextrose (SD), with the final amino acid concentrations as follows: adenine [10 μg/mL], uracil [20 μg/mL], tryptophan [20 μg/mL], histidine [20 μg/mL], arginine [20 μg/mL], methionine [20 μg/mL], tyrosine [30 μg/mL], leucine [60 μg/mL], isoleucine [30 μg/mL], lysine [30 μg/mL], phenyalanine [50 μg/mL], glutamate [100 μg/mL], aspartate [100 μg/mL], valine [150 μg/mL], threonine [200 μg/mL], serine [200 μg/mL]. These plates contain low adenine and are referred to as SDClowA plates. All cell growth was performed at 30°C except where otherwise noted.

The color from the *ade3–2p* reporter was most consistent when SDClowA plates were poured in a precise manner three days prior to use. For a 2 L batch, the following was added together in a 4 L flask: 13.4g Yeast Nitrogen base without amino acids, 40g Bacto-agar, 1 stir bar, and 1850mL MQH_2_O. The opening of the flask was covered with foil, and the mix was stirred for 5 min. Media was autoclaved on a liquid cycle (30 min at 121°C with slow exhaust) in a dry autoclavable plastic tray. To prevent excessive heating the flask was removed as soon as the jacket pressure allowed the door to open, and the media was stirred for 10 min to mix and cool. At this point powdered amino acid mix and 100mL 40% Dextrose were added. The media then stirred for an additional 10 min to further mix and cool. Plates were poured using a PourBoy 4 plate pouring machine to dispense 33 mL of media per plate. These SDClowA plates were stacked unwrapped to allow them to dry but shielded from light until use in the assay.

For microscopy, cells were imaged in SDC-Trp media to maintain selective pressure on the integrated GFP-Tub1 construct. Prior to imaging, cells were grown and induced in S media supplemented with 3% wt/vol raffinose + 0.05% wt/vol dextrose (to form SRd). Amino acid concentrations for both medias are as follows: adenine [40 μg/mL], uracil [40 μg/mL], tryptophan [0 μg/mL], histidine [40 μg/mL], arginine [40 μg/mL], methionine [40 μg/mL], tyrosine [60 μg/mL], leucine [120 μg/mL], isoleucine [60 μg/mL], lysine [60 μg/mL], phenyalanine [100 μg/mL], glutamate [200 μg/mL], aspartate [200 μg/mL], valine [300 μg/mL], threonine [400 μg/mL], serine [400 μg/mL].

### Re-replication induction strategy

Re-replication was conditionally induced in a strain background where *ARS317* preferentially reinitiates [4]. The strain background was deregulated in three ways: (1) (*MCM7–2NLS*)—the CDK-driven export of Mcm2–7 from the nucleus [74,79,80] was blocked by fusing a constitutive nuclear localization signal onto the endogenously expressed Mcm7; (2) (*pGAL-∆ntcdc6-cdk2A*)—the CDK inhibition of Cdc6, which occurs through transcriptional regulation [81], phosphorylation-directed degradation [82–84], and direct CDK binding [85], was disrupted by expressing an extra copy of Cdc6 lacking CDK phosphorylation and binding sites under a galactose-inducible promoter; and (3) (*orc6(S116a)*)—the CDK inhibition of ORC by phosphorylation of Orc2 and Orc6 was constitutively yet minimally perturbed by eliminating one of four CDK consensus phosphorylation sites on Orc6 [6].

To induce re-replication, galactose is added to express *∆ntcdc6-cdk2A* from the *pGAL* promoter. This increases the G2 DNA copy number of *ARS317* by 50% from 2C to 3C. Despite the constitutive deregulation of Mcm7 and Orc6, no detectable re-replication could be detected by microarray comparative genomic hybridization (our most sensitive copy number assay) in the absence of *∆ntcdc6-cdk2A* induction. Nonetheless, in principle cryptic and sporadic re-replication events could occur throughout the genome before the induction. To focus on the consequences of re-replication specifically induced at *CEN5* from *ARS317*, we use congenic strains either lacking *ARS317* at *CEN5* or with *ARS317* located on the arm of the chromosome as controls for colony sectoring frequency and aneuploid frequency.

### Sectoring assay following re-replication induction

Colony color development and sectoring frequencies were most reproducible when freshly thawed cells were used and the re-replication induction and plating were performed in a precise manner. Yeast were thawed from frozen glycerol stocks onto YEPD plates and grown at 30°C. The following day, this patch was used to inoculate 25 mL of YEPD and was grown to an OD_600_ between 0.4 and 0.5 over the course of 4–5 hours at 30°C in a shaking (250 rpm) waterbath. From this culture, we inoculated the experimental culture grown at 30°C in non-repressive rich media containing 3% raffinose and 0.05% dextrose (YEPRd) so that after 13–15 hours, the culture would be growing exponentially at an OD_600_ between 0.4 and 0.5.

To arrest cells, nocodazole (US Biological, Salem, MA; Cat. no. N3000) was added to the culture at a final concentration of 15 μg/mL. After two hours of incubation, effective mitotic arrest was confirmed microscopically (>90% large budded cells) and 40% galactose was added to the arrested culture to a final concentration of 2.7%. Galactose induction was allowed to proceed for three hours before cells were washed and plated. A modification of this assay was used for YJL9631 and YJL9633, which have *ARS317* integrated at the right end of Chromosome V. Total reinitiation from the right end of Chromosome V in these strains was higher after 3 hours of induction than the centromeric reinitiation from strains with *ARS317* integrated near *CEN5*, possibly from the low-level endogenous re-replication occurring on the end of the arm. Thus, to compare the consequences of equivalent amounts of reinitiation at the two locations, we reduced the galatose induction time for YJL9631 and YJL9633 to two hours. This was done by delaying the galatose induction until 3 hours after addition of nocodazole so that all strains were exposed to nocodazole for the same amount of time (5 hr). Cell cycle arrest and maintenance of arrest during the induction of re-replication were confirmed by flow cytometry.

After the induction, cells were diluted into YEPD to an estimated concentration of 1000 CFU/mL based on (1) the OD_600_ of the culture at the time of nocodazole addition (using a conversion factor of 2 x 10^7^/mL at an OD_600_ = 1.0) and (2) the expected viability drop following the induction of re-replication (see below). From this dilution, 200μL (~200 CFU) were promptly spread onto SDClowA plates using 6–8 sterile glass beads per plate. The plates were incubated face-up at 30°C in the middle of an air incubator for 5–7 days, after which the plates were placed into a dark drawer at room temperature (18–22°C) for further color development. After 2–3 days there was optimal distinction between red and pink colors, and the plates were scored for color-sectored colonies.

After the total number of colonies was counted, plates were manually screened using a Leica Modular Stereomicroscope MZ6 and a Leica KL1500 LCD 150-watt halogen cold light source and ring-light fixture. Lamp was set to 3200 K with the aperture 80% open. To be scored, sectored colonies were required to be at least 1 mm in diameter and have a red portion that was less than 75% but more than 25% of the total colony. This range of sector sizes was chosen to accommodate differences in the time the two daughter cells might take to recover from the cell cycle arrest or possible differences in doubling times for the two daughter lineages. We were stringent about sector color, however. The pink portion of each red/pink colony had to have the same tint as the surrounding pink colonies (indicative of the color a single copy of *ade3–2p* produced), and the red portion had to be of an unmistakably darker tint than the pink portion. The white portion had to be completely white and without colored tint. Colonies containing more than two colors were not scored. All sectors had to originate from the center of the colony, indicating they had been formed in an early cell division immediately following centromeric re-replication. Colonies with colored sectors originating from outside the center were not scored. The results of all trials reported in this study are listed in S7 Table.

After all plates were screened and sectors tallied, colonies were picked from their original plate and struck onto a new SDClowA plate, then incubated as described above to develop color. This colony purification process allowed us to obtain clonal isolates from each sector of the colony. From the streak, individual colonies were picked (one of each color) and patched onto rich media with 2% dextrose. These patches provided the material for glycerol freezer stocks, kept in 96-well plates at—80°C.

The viability drop during the plating described above was determined in preliminary experiments by plating out an estimated 200 CFU per plate based solely on the OD_600_, then measuring the actual number of colonies appearing. Measured and estimated colony numbers were the same (within 10–15%) for cells that were arrested in nocodazole for 2 hr, indicating that there was little viability drop at the T = 0 hr time point. After a 3 hr induction of re-replication (T = 3 hr), however, there was an ~4x drop in viability due in large part to the prolonged exposure to nocodazole (5 hr). Importantly, this viability drop was not dependent on the presence or absence of *ARS317* near *CEN5*, whereas the sectoring frequency among the surviving colonies was. We thus inferred that this frequency was independent of the viability drop and was representative of the sectoring frequency that the entire population would have displayed in the absence of a viability drop. Finally, we note that the T = 3 hr viability drop was somewhat different in the recombination mutants we examined: ~6x for *rad52*∆ and *rad52∆ dnl4∆*, and ~3x for *dnl4∆*.

### Statistical analysis of sectoring frequencies

To determine if the mean red/white-colony or red/pink-colony frequencies after centromeric re-replication was significantly higher than that of the no-*ARS317* control (Fig. 2 & Fig. 3A), a one-tailed, two-sample *t*-test was applied using Excel. To determine whether the mean red/pink-colony frequencies after centromeric re-replication in recombination mutant backgrounds (*rad52∆*, *dnl4∆*, *rad52 dnl4∆*) were significantly different than in wild-type recombination backgrounds a two-tailed, two-sample *t*-test was applied using Excel. All sectoring frequencies used in the *t*-tests are listed in S7 Table. All *p* values, regardless of significance, are reported in the appropriate figure legends. Asterisks used in the figures indicate level of confidence, with three (***) indicating a *p*-value of <0.001, and two (**) indicating a *p*-value between 0.01 and 0.001.

### Monitoring re-replication via aCGH

The remainder of the cells not used in the platings described above were harvested after the induction of re-replication and their DNA extracted as previously described (referred to as “method 2”) [18]. We have referred to this protocol as the “Clean Genomic Prep” on the GEO microarray database. This DNA was hybridized against reference DNA from YJL8590 (collected from a nocodazole-arrested culture that did not undergo re-replication) in the manner described previously [4]. Briefly, reference DNA was labeled with Cy5 fluorescent dye and DNA from the re-replicating strain was labeled with Cy3 fluorescent dye. Equal quantities were combined and competitively hybridized to an in-house printed microarray containing approximately 13,000 genic and intergenic PCR product elements representing the entire *S*. *cerevisiae* genome (GEO platform number GPL3412) at 63°C for at least 20 hours. Arrays were then scanned using an Axon Scanner 4B and analyzed as described previously [4]. All microarray data is deposited in the Gene Expression Omnibus [86] (http://www.ncbi.nlm.nih.gov/geo/) with accession number GSE55641.

### Copy number determination via aCGH

Sectored colonies were chosen at random for analysis of copy number across the genome. For each such colony both purified sectors were thawed from the glycerol freezer plate stock onto YEPD plates and grown at 30°C overnight. A part of each patch was then used to inoculate 5 mL liquid YEP8D, which was grown to saturation over 2–3 days at 30°C with most cells arrested in G0 phase.

To obtain the DNA, the extraction method used in the re-replication DNA extraction was simplified to handle numerous small cultures at once. This DNA protocol is referred to as the “Small DNA Prep” on the GEO microarray database. 1 mL from a saturation culture was moved to a 2 mL screw-capped tube and spun down in a Eppendorf 5417C microfuge at 14,000 rpm for 3 min. Cell pellets were washed twice with 1 mL water and spun down at 14,000 rpm for 3 min for each wash. Cell pellets were then flash-frozen in liquid nitrogen and either processed immediately or stored at—80°C for future extraction. To the frozen cell pellets, 200 μL lysis buffer (2% Triton X-100, 1% SDS, 100 mM NaCl, 10 mM TrisCl pH 8, 20 mM EDTA) was added and tubes were allowed to rock gently at 4°C for 10 min to mix in the buffer as well as thaw the pellet. Then, 400 μL small glass beads and 200 μL phenol:chloroform:isoamyl alcohol (25:24:1) were added to the tube and vortexed immediately to mix. Tubes were vortexed on high for 10 min in a Vortex genie, after which 400 μL 1X TE (10 mM TrisCl pH 7.5, 1 mM EDTA) and 400 μL phenol:chloroform:isoamyl alcohol (25:24:1) were added. The tubes were vortexed to mix then spun down at 13300 rpm for 10 min in the microfuge on soft. 500 μL of the clear upper aqueous phase was transferred to new screw-capped tubes, containing 500 μL chloroform:isoamyl alcohol (24:1), and vortexed well. Tubes were spun at 5100 rpm for 10 min in the microfuge, and 450 μL of the top phase was moved to a new microfuge tube. Volumes were brought up to 500μL with 50μL 1x TE pH 7.5, and 400 μL isopropanol and 5 μL 5 M NaCl were added followed by vortexing. Tubes were spun at 10600 rpm for 10 min in the microfuge. The supernatant was carefully aspirated out of microfuge tubes, and the pellet was washed with 750 μL 70% ethanol by vortexing well. Tubes were spun at 10600 rpm for 5 min in the microfuge and the supernatant was carefully aspirated out. DNA pellets were dried for 10 min with gentle heat in a speedvac. Pellets were resuspended in 175 μL 1x TE pH 7.5 by heating in a 37°C waterbath with vortexing for 15 min. 1 μL RNase (Qiagen, Cat. no. 19101, DNase free, 100mg/mL) was added to each tube, which were inverted to mix and incubated in the 37°C waterbath for 1 hour. After the incubation, 100 μg Proteinase K (Roche, Cat. no. 03115852001) was added to each tube and inverted to mix, then placed in a 55°C waterbath to incubate for 30 min. Then, 400 μL chloroform:isoamyl alcohol (24:1) was added to each tube followed by vortexing and a spin at 7500 rpm for 10 min in the microfuge. The top 150 μL was removed and added to new microfuge tubes, followed by 120 μL isopropanol and 1.5 μL 5 M NaCl. Tubes were vortexed and spun at 9600rpm for 10 min in the microfuge. The supernatant was carefully aspirated out of microfuge tubes, and the pellet was washed with 750 μL 70% ethanol by vortexing well. Tubes were spun at 9600 rpm for 5 min in the microfuge and the wash was carefully aspirated out. DNA pellets were dried for 10 min with gentle heat in a speedvac. Pellets were dissolved in 50 μL 2 mM Tris pH 7.8 and placed in the 37°C waterbath for 15 min to ensure complete resuspension. DNA was then kept at—20°C until used for aCGH.

DNA from the sector isolates was hybridized against reference DNA from YJL8590 (collected from a nocodazole-arrested culture that did not undergo re-replication) in the manner described previously [4]. Briefly, reference DNA was labeled with Cy5 fluorescent dye and DNA from the color sector was labeled with Cy3 fluorescent dye. Equal quantities were combined and applied to an in-house printed microarray (GEO platform number GPL3412) and hybridized at 63°C for at least 20 hours. Arrays were then scanned using an Axon Scanner 4B and analyzed as described previously to generate copy number information across the genome [4]. All microarray data is deposited in the Gene Expression Omnibus [86] (http://www.ncbi.nlm.nih.gov/geo/) with accession number GSE55641.

To interpret the copy number of Chromosome V in the red or white sectors, we had to take into account the fact Chromosome V monosomy and trisomy are not stable in the long term. Populations of diploid cells containing these aneuploidies are eventually taken over by cells that have become disomic for Chromosome V. Hence, during the growth, freezing, and thawing of these aneuploidy cells, the population average of Chromosome V copy number gradually declines from 3C to 2C (for trisomy) or increases from 1C to 2C (for monosomy). In contrast, the populations of cells that start off euploid with a 2C copy number of Chromosome V never show any significant difference from 2.00 C during continued growth. Hence, we scored red sectors with Chromosome V copy number > 2.2C as trisomic for Chromosome V and white sectors with Chromosome V copy number < 1.8C as monosomic for Chromosome V. Both criteria had to hold in order for a red/white colony to be scored as a 2:0 segregation event. For red/pink colonies to be scored as a 2:1 segregation event, Chromosome V copy number in the red sectors had to be > 2.2C and in the pink sector at 2.00 C.

During our copy number analysis we discovered several sectored colonies, in which the Chromosome V copy number in the red and/or white sectors was significantly different than 2.00C, but not different enough to satisfy the thresholds of > 2.2C and < 1.8C respectively. Because we suspected that the stress of freeze-thawing placed selective pressure on the outgrowth of euploid cells, we isolated DNA directly from the frozen cells to perform aCGH. Cells from the frozen stock were placed directly in a screw-cap tube with 1 mL water, then processed the same as described for saturation cultures. In almost all of these cases, the new copy number analysis established that the red sectors had a much greater than 2.2C copy number for Chromosome V, while the white sectors had a much less than 1.8C copy number for Chromosome V.

### Live-cell microscopy: Cell growth and re-replication induction

Cell growth and re-replication induction were performed in a similar manner as the re-replication assays described above. Yeast were thawed from frozen glycerol stocks onto SDC-Trp plates and grown at 30°C. The following day, this patch was used to inoculate 25 mL of SDC-Trp and was grown to an OD_600_ between 0.2 and 0.4 over the course of 6 hours at 30°C in a shaking (250 rpm) water bath. From this culture, we inoculated the experimental culture grown at 30°C in non-repressive rich media containing 3% raffinose and 0.05% dextrose (SRd) so that after 13.5 hours, the culture would be growing exponentially at an OD_600_ between 0.3 and 0.5 and be amenable to rapid galactose induction.

To monitor the exertion of metaphase spindle tension on re-replicated centromeres, we could not pre-arrest cells in metaphase with nocodazole before inducing re-replication (as outlined in Fig. 1A). Instead, we took advantage of the fact that re-replication itself induces a metaphase arrest with an active mitotic spindle because of the DNA damage response triggered by re-replication [18, 26]. Hence, we induced re-replication in exponentially growing cells by adding 40% galactose to a final concentration of 2.7%. For video microscopy (Fig. 5C; S1 and S2 Movies), galactose induction was allowed to proceed for approximately 3 hours, after which a small sample (500 μL) was removed and imaged live (see “Video microscopy” below). For time-lapse imaging (Figs. 5A and 5B), cultures were washed by vacuum filtration using 10 volumes of pre-warmed sterile water, then resuspended in 25 mL pre-warmed SDC-Trp media to limit further galactose induction of re-replication. From this new resuspension, a small sample (500 μL) was imaged continuously for approximately 20 min before nocodazole was added (see “Time-lapse imaging before nocodazole treatment”below) and then for approximately 2 hours after nocodazole was added (see “Time-lapse imaging after nocodazole treatment” below).

### Live-cell microscopy: Imaging re-replicated cells

All live imaging was conducted in a temperature-controlled chamber maintained at 30°C with yeast immobilized to the bottom of a chambered coverslip (Lab-Tek/Thermo Fisher, Cat. no.12565401). Briefly, the bottom of the chamber was coated using 100 μL of 0.5 μg/mL Concanavalin A Type IV (Sigma-Aldrich, Cat. no. C2010–25MG) and allowed to air dry in the dark at 30°C for 1 hour. Roughly 3.5x10^6^ cells in 500 μL was pipetted into the chamber and allowed to sit for 15 min at 30°C, after which the chamber was gently washed twice to remove excess cells. Chambers were then imaged on the Deltavision deconvolution microscope (Applied Precision) using SoftWorx image acquisition software.

**Video microscopy**. Re-replicating cell spindle and spot dynamics (Fig. 5C; S1 and S2 Movies) were visualized with a 100× 1.40 UPLS Apo objective (Olympus) and a CoolSNAP_HQ / ICX285 camera. To visualize DNA, Hoechst 33342 stain was added at a final concentration of 10 μg/mL to the cells prior to imaging. A set of three images (one for each channel) was taken every 6 seconds for 10 min; the Z plane remained unchanged and was prevented from drifting using the UltimateFocus feature. The settings for each excitation wavelength are as follows: RD-TR-PE (red channel; ex: 555 nm, em: 617 nm; 32% power; 0.6 s exposure), FITC (green channel; ex: 490 nm, em: 528 nm; 50% power, 1 s exposure), and DAPI (blue channel; ex: 360 nm, em: 457 nm; 32% power; 0.2 s exposure).

**Time-lapse imaging before nocodazole treatment**. Spot and spindle visualization before and after nocodazole (Fig. 5A) was imaged with a 60× 1.42 NA Plan Apo objective (Olympus) and a CoolSNAP_HQ / ICX285 camera. No Hoechst stain was added to these samples. X, Y, and Z coordinates were recorded for 25 locations on the chambered coverslip, 10 for the no *ARS317* strain and 15 for the *ARS317* at *CEN5* strain. For each of these points, a Z-stack was taken ±1 μm from the recorded Z coordinate in steps of 0.5 μm for both the red and green channels. This recorded Z coordinate was prevented from drifting using the UltimateFocus technology. Four stacks were acquired for each marked position as quickly as possible, resulting in images taken every 7 min for nearly 20 min. The settings for each excitation wavelength are as follows: RD-TR-PE (red channel; ex: 555 nm, em: 617 nm; 32% power; 0.8 s exposure), and FITC (green channel; ex: 490 nm, em: 528 nm; 50% power, 1 s exposure).

**Time-lapse imaging after nocodazole treatment**. Media containing nocodazole was carefully added to the chambers with a curved pasteur pipet so as not to disturb chamber position on the microscope stage. Immediately before addition, nocodazole was added to 500 μL fresh SDC-Trp media and then dripped into each chamber for a final nocodazole concentration of 75 μg/mL. Nocodazole was kept separate from the media until the last minute to minimize its precipitation out of the aqueous media. Using the same 25 points as the pre-nocodazole imaging, a Z-stack was taken ±1 μm from the recorded Z coordinate in steps of 0.5 μm for both the red and green channels. This recorded Z coordinate was prevented from drifting using the UltimateFocus technology. Four stacks were acquired for each marked position every 10 min for 2 hours. The settings for each excitation wavelength are as follows: RD-TR-PE (red channel; ex: 555 nm, em: 617 nm; 32% power; 0.8 s exposure), and FITC (green channel; ex: 490 nm, em: 528 nm; 50% power, 1 s exposure).

### Live-cell microscopy: Spot analysis of re-replicated cells

**Quantification of spot number before nocodazole treatment**. All images were assigned with a random numerical prefix using the free demo version of Renamerox (Branox Technologies) so that they may be scored blindly. Using the free imaging software Fiji [87], a single Z-stack was opened and cells meeting specific criteria were annotated with a letter. The criteria are as follows: the cell must remain large-budded with a short (≤ 2 μm) spindle indicating a stable metaphase arrest without entry into anaphase. Using the Z- and time-dimension, the number of centromeric spots for each cell satisfying the criteria was determined and recorded. Centromere-breathing and rapid movement of spots across Z sections made it necessary to look through all sections of a Z-stack and through all four time frames during our assessment of the number of resolvable spots (see S6 Table).

It should be noted that roughly 8% of cells scored contained spots with noticeable differences in intensity by eye (our most sensitive way of detecting these differences). Such difference could conceivably arise from a number of factors, including: (1) the presence of overlapping unresolved centromeres in some spots; (2) differences in how close the Z sections cut through the center of each spot; (3) possible lag times in restoring complete binding of fluorescent proteins after their displacement by re-replication. The 8% of cells we flagged were those showing spot intensity differences that persisted through multiple pre-nocodazole time frames.

Within these cells, we were particularly interested in determining how many spots likely represented two (or more) centromeres that were never resolved in any time frame, as this would give us an indication of the extent to which centromeres were undercounted. To do this, we looked for spots whose intensity was more than 170% of a neighboring spot within the same cell. Quantification of spot intensity was performed using Fiji. Because of high signal to noise ratio, large variability in the noise for different Z sections, and movement of spots among the Z sections, each spot was quantified using the Z section and time point where the intensity of the spot was greatest. The total pixel intensity for a fixed-size circle was measured both when the circle was centered over the spot and when the circle was placed just outside the spot. The difference was then calculated to determine the spot intensity corrected for the neighboring noise. We note that the surrounding noise could be quite different for the different spots in the same cell, as it was lower for spots near the periphery of the cell body (e.g. within the bud neck) and was higher for spots closer toward the center. Based on this quantification of spot intensity, the spots that potentially represented two or more unresolved centromeres comprised only 2–3% of the total number of spots we counted. Because of this low proportion and the challenges described above of accurately quantifying spot intensity, we decided to adhere to the accepted practice in the field of counting the number of fluorescently marked chromosomal spots without adjusting for spot intensity. Nonetheless, this trial quantification provided further indication that we were not significantly undercounting the number of centromeres following their induced re-replication.

**Quantification of spot number before nocodazole treatment**. All images were assigned with a random numerical prefix using the free demo version of Renamerox (Branox Technologies) so that they could be scored blindly. Using the imaging software Fiji [87], single Z-stacks were opened until the file matching the pre-nocodazole treatment was found. Then, using the annotated pre-nocodazole image as a guide, each cell was revisited after 2 hours of nocodazole treatment. At this specific timepoint, the Z-planes were examined to determine how many spots were present. Several spots that were originally scored in the pre-nocodazole treatment were discarded due to one of the following reasons: the cell underwent anaphase before the nocodazole treatment had an effect; the cell’s spots separated into opposite lobes after spindle disappearance, indicating they were mid-anaphase; the spindle was not broken down; the centromeric spot was not visible at the final 2 hour timepoint, either because it lost signal or it became out of focus; or the cell died. Thus only large-budded cells in metaphase prior to nocodazole with spindle breakdown and visible spot(s) 2 hours after nocodazole were scored. For each strain in each trial, ≥ 100 cells were scored. Values were recorded and can be seen graphically in Fig. 5B or in S6 Table.

It should be noted that all images were scored with only moderate and uniform changes to brightness and contrast as needed.

### Image creation for Fig. 5, S6 Fig, and S1 and S2 Movies

For images viewed in Fig. 5A and S6 Fig, the entire images were adjusted uniformly for brightness and contrast only.

For images viewed in Fig. 5C and S1 and S2 Movies, the red channel of this montage was subjected to Fiji’s bleach correction plugin using an exponential line fit. The resulting image fit a curve with R^2^ > 0.99. To remove background, it was subtracted using Fiji’s background subtraction, using a rolling ball radius of 200 pixels. The green channel was altered only in brightness and contrast. The blue channel underwent a simple ratio (0.5) bleach correction using Fiji’s bleach correction plugin.

## Supporting Information

S1 Fig Red/pink colony frequencies induced by re-replication in strains lacking the reinitiating origin *ARS317* and deficient in recombinational repair. Diploid re-replicating strains with no *ARS317* on the *ade3–2p* marked Chromosome V homolog and containing homozygous deletions of the indicated genes were scored for the frequency of red/pink sectored colonies both before (–) and after (+) re-replication as described in Fig. 3 (see S3 Table). Data is presented as the mean ± SD (n ≥ 3).
(TIF)

S2 Fig Re-replication profile of Chromosome V for diploid strains deficient in recombinational repair. Diploid re-replicating strains with homozygous deletions of indicated genes and both reinitiating origin *ARS317* and *ade3–2p* integrated at *CEN5* (circle) were arrested in metaphase and induced to re-replicate for 3 hr as described in Fig. 1A (see S1 Table). DNA copy number was analyzed by array CGH with baseline normalized to 4C.
(TIF)

S3 Fig Red/white sectoring frequencies for strains deficient in recombinational repair. Diploid re-replicating strains with homozygous deletions of indicate genes and *ade3–2p* integrated at *CEN5* were induced to re-replicate as described in Fig. 1A were scored for the frequency of red/white sectored colonies either before (-) or after (+) a 3 hr induction of re-replication (see S3 Table). Data is presented as the mean ± SD (n ≥ 3). (A) Strains containing no *ARS317* (YJL9627). (B) Strains containing *ARS317* integrated at *CEN5* (YJL9637).
(TIF)

S4 Fig Re-replication profiles of Chromosome V for diploid strains induced to re-replicate for 3 hr without prior metaphase arrest. *ARS317* and *ade3–2p* mark integration sites of the reinitiating origin and the copy number reporter, respectively. Inset shows schematic of re-replication bubbles inferred from profiles. Circles on X-axis and in schematic represent centromere *CEN5*. Upper panel: YJL9637. Lower panel: YJL9627.
(TIF)

S5 Fig Cycling cells induced to re-replicate transiently arrest in M phase. Flow cytometry of strains analyzed in S4 Fig before and after the induction of re-replication. (A) Strain containing no *ARS317* (YJL9627). (B) Strain containing *ARS317* integrated at *CEN5* (YJL9637).
(TIF)

S6 Fig Dynamic separation and movement of re-replicated centromeres depends on mitotic spindle tension. Centromeric re-replication was induced for 3 hr in asynchronously growing cells containing tdTomato marked centromeres and GFP tagged tubulin (YJL10671). Following the induction, which arrested cells in metaphase, time-lapse Z-stack images were taken every 7 min over approximately 20 min. Nocodazole was then added to eliminate the mitotic spindle, and imaging was continued every 10 min for 2 hr. Each row shows representative time-lapse images taken from a single cell either before nocodazole addition or after.
(TIF)

S1 Table Array CGH for the re-replication profiles presented in this work. (DOCX)

S2 Table Array CGH results corresponding to Fig. 2. Chromosomes other than ChrV are at a copy number of 2.0 unless listed in “Other genomic changes” with copy number reported in parentheses. For chromosomal segments with a copy number other than 2.0, the boundaries of the segments are indicated by chromosomal coordinates within brackets. We inferred that the *ade3–2p* marked ChrV homolog had undergone a 2:0 segregation event if the total ChrV copy number was > 2.2 in the red sector and < 1.8 in the white sector (see Materials and Methods). LT = left telomere; RT = right telomere
(DOCX)

S3 Table Array CGH results corresponding to Fig. 3. Chromosomes other than ChrV are at a copy number of 2.0 unless listed in “Other genomic changes” with copy number reported in parentheses. For chromosomal segments with a copy number other than 2.0, the boundaries of the segments are indicated by chromosomal coordinates within brackets. We inferred that the *ade3–2p* marked ChrV homolog had undergone a 2:1 segregation event if the total ChrV copy number was > 2.2 in the red sector and = 2.0 in the pink sector (see Materials and Methods). LT = left telomere; RT = right telomere; Mix = whole copy number of ChrV cannot be reported due to segmental gains or losses.
(DOCX)

S4 Table Array CGH results corresponding to Fig. 4B. Chromosomes other than ChrV are at a copy number of 2.0 unless listed in “Other genomic changes” with copy number reported in parentheses. For chromosomal segments with a copy number other than 2.0, the boundaries of the segments are indicated by chromosomal coordinates within brackets. We inferred that the *ade3–2p* marked ChrV homolog had undergone a 2:0 segregation event if the total ChrV copy number was > 2.2 in the red sector and < 1.8 in the white sector (see Materials and Methods). LT = left telomere; RT = right telomere; Mix = whole copy number of ChrV cannot be reported due to segmental gains or losses.
(DOCX)

S5 Table Array CGH results corresponding to Fig. 4D. Chromosomes other than ChrV are at a copy number of 2.0 unless listed in “Other genomic changes” with copy number reported in parentheses. For chromosomal segments with a copy number other than 2.0, the boundaries of the segments are indicated by chromosomal coordinates within brackets. We inferred that the *ade3–2p* marked ChrV homolog had undergone a 2:1 segregation event if the total ChrV copy number was > 2.2 in the red sector and = 2.0 in the pink sector (see Materials and Methods). LT = left telomere; RT = right telomere; Mix = whole copy number of ChrV cannot be reported due to segmental gains or losses.
(DOCX)

S6 Table Scoring fluorescent spots marking *CEN5* before and after nocodazole treatment. After induction of re-replication, the number of resolvable spots in each cell was counted before and after nocodazole treatment (see Materials and Methods). Cells with the same number of spots before nocodazole treatment were grouped together and each group was then categorized based on the number of spots that could still be resolved after nocodazole treatment. Percentages are based on the total number of cells scored for the indicated initial spot number.
(DOCX)

S7 Table Results of all re-replication assays reported in this work. Colony counts and sector frequencies are shown for each experimental trial. Mean frequencies (not weighted for trial size), standard deviations, and *p*-values from *t*-test statistical analyses (see Materials and Methods) were calculated from these sector frequencies.
(DOCX)

S8 Table Calculation of segregation frequencies. Mean sectoring frequency for each set of repeated re-replication trials (n ≥ 3) was multiplied by the fraction of sectored colonies that exhibited the distribution of ChrV copy number expected for the relevant segregation event (see Materials and Methods).
(DOCX)

S9 Table Strains used in this study. For each locus, the allele of the *MATa* parent is listed first. Key features of each strain are in bold.
(DOCX)

S10 Table Flanking homologies for integration of re-replication and gene deletion fragments. Cassettes and fragments were targeted for homologous recombination into the genome (see Materials and Methods) by flanking sequences mapping to the left and right as determined by the conventional left to right polarity of each chromosome. Sequence is reported in the 5’ to 3’ direction of the Watson strand of the S288C reference sequence from the Saccharomyces Genome Database (version R64–1–1).
(DOCX)

S11 Table Oligonucleotides used to PCR fragments for strain construction. Uppercase indicates where oligo hybridizes to the PCR template; lowercase corresponds to chromosomal sequences used to target integration of fragment (see Materials and Methods).
(DOCX)

S1 Movie Split-channel spot-spindle dynamics. The full-length movie for the montage presented in Fig. 5C was split by color channel and combined side-by-side to highlight the dynamic movement of the individual components. Left movie: spots corresponding to *tet* operator arrays bound to tdTomato-tagged Tet repressors (on the left of *CEN5*); center movie: the spindle (Tub1-GFP); right movie: DNA stained with Hoechst.
(AVI)

S2 Movie Composite spot-spindle dynamics. The full-length movie for the montage presented in Fig. 5C with all three channels overlaid. Red: spots corresponding to *tet* operator arrays bound to tdTomato-tagged Tet repressors (on the left of *CEN5*); green: the spindle (Tub1-GFP); blue: DNA stained with Hoechst.
(AVI)
