# Supplementary figures and images for: Re-replication of a Centromere Induces Chromosomal Instability and Aneuploidy
Source: PLoS Genet. 2015 Apr 22;11(4):e1005039. doi: 10.1371/journal.pgen.1005039 (PMC4406714; doi:10.1371/journal.pgen.1005039)

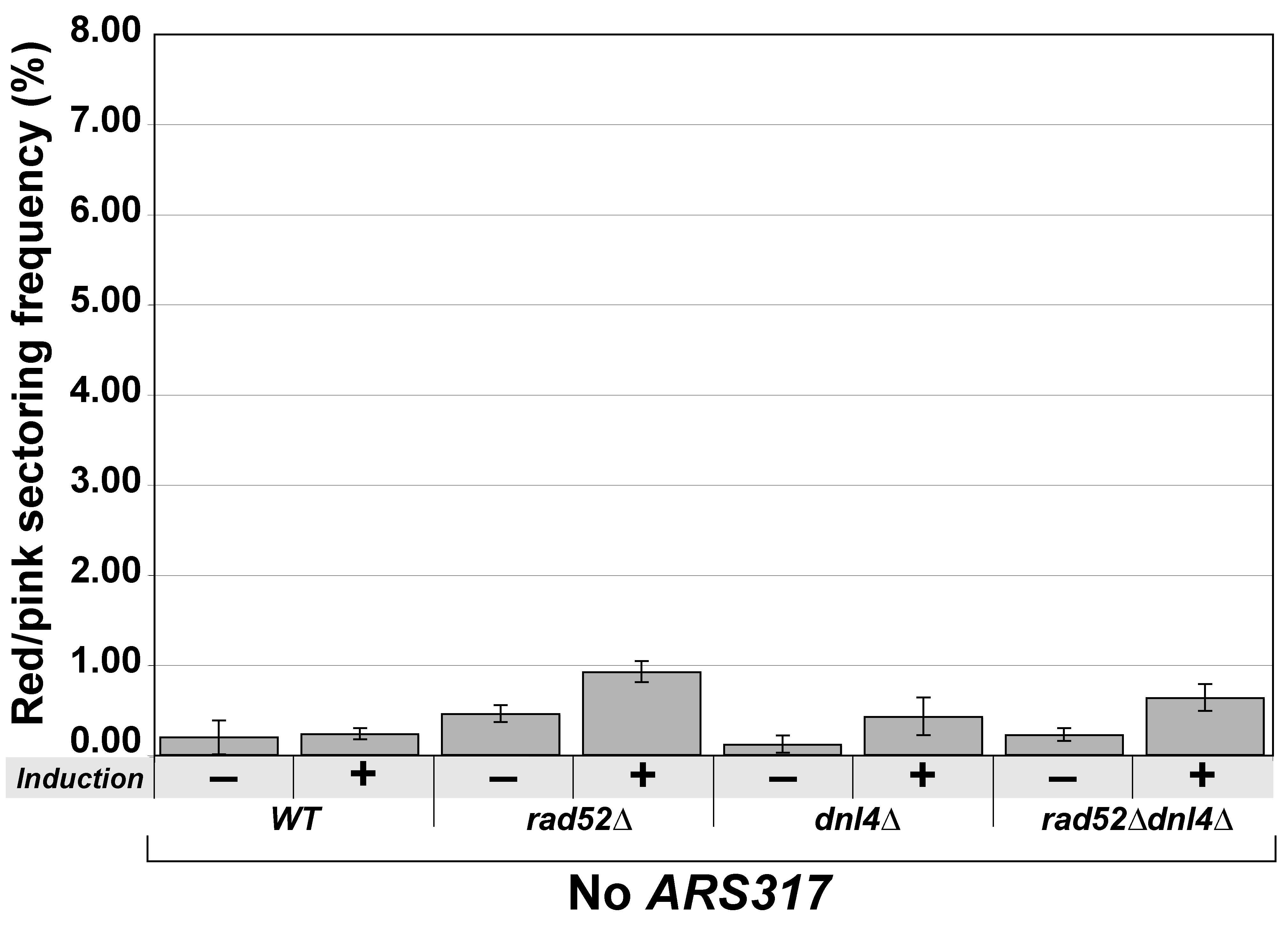

Supplement: S1 Fig — Diploid re-replicating strains with no ARS317 on the ade3–2p marked Chromosome V homolog and containing homozygous deletions of the indicated genes were scored for the frequency of red/pink sectored colonies both before (–) and after (+) re-replication as described in Fig. 3 (see S3 Table). Data is presented as the mean ± SD (n ≥ 3). (TIF) [file pgen.1005039.s001.tif]

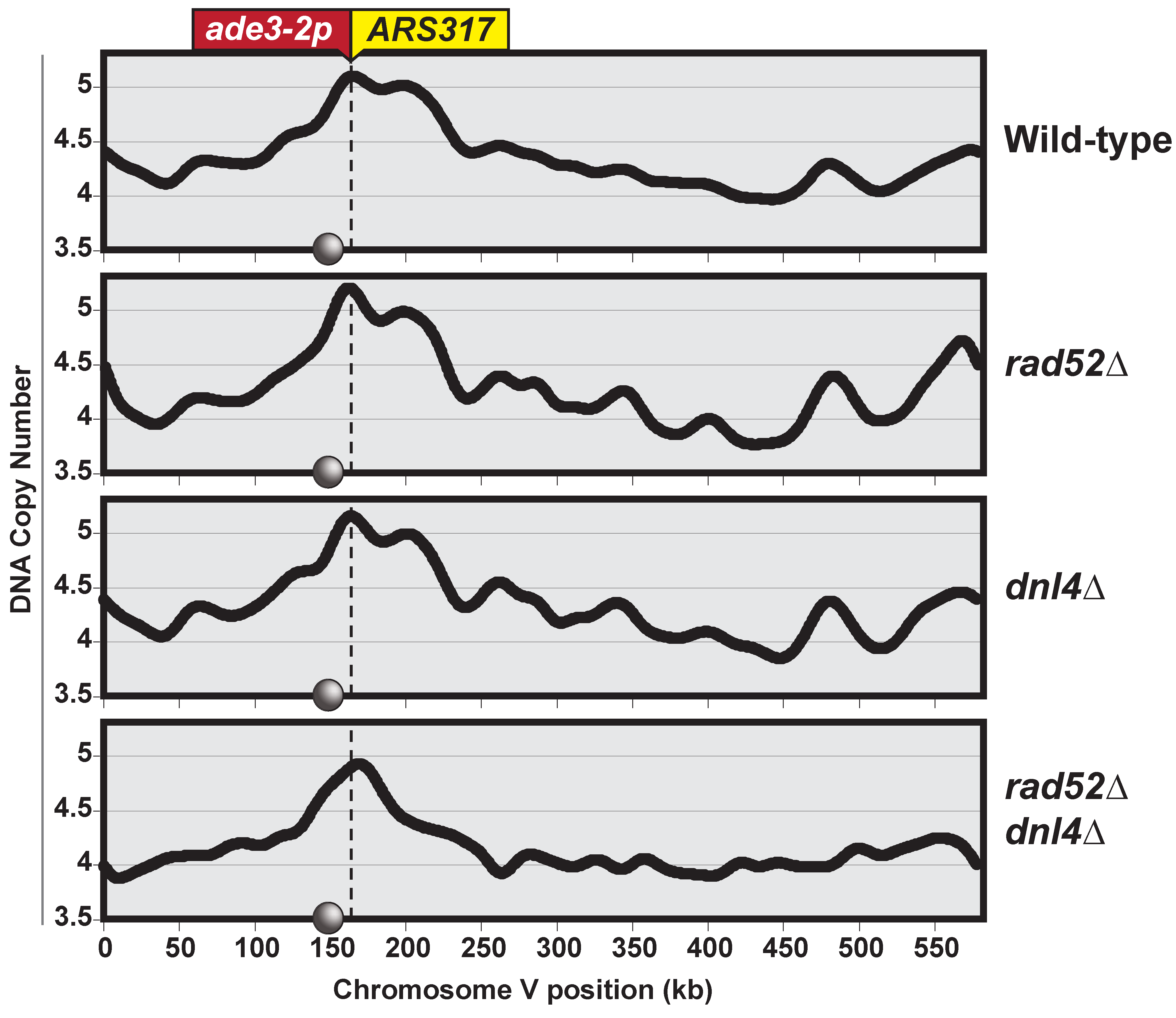

Supplement: S2 Fig — Diploid re-replicating strains with homozygous deletions of indicated genes and both reinitiating origin ARS317 and ade3–2p integrated at CEN5 (circle) were arrested in metaphase and induced to re-replicate for 3 hr as described in Fig. 1A (see S1 Table). DNA copy number was analyzed by array CGH with baseline normalized to 4C. (TIF) [file pgen.1005039.s002.tif]

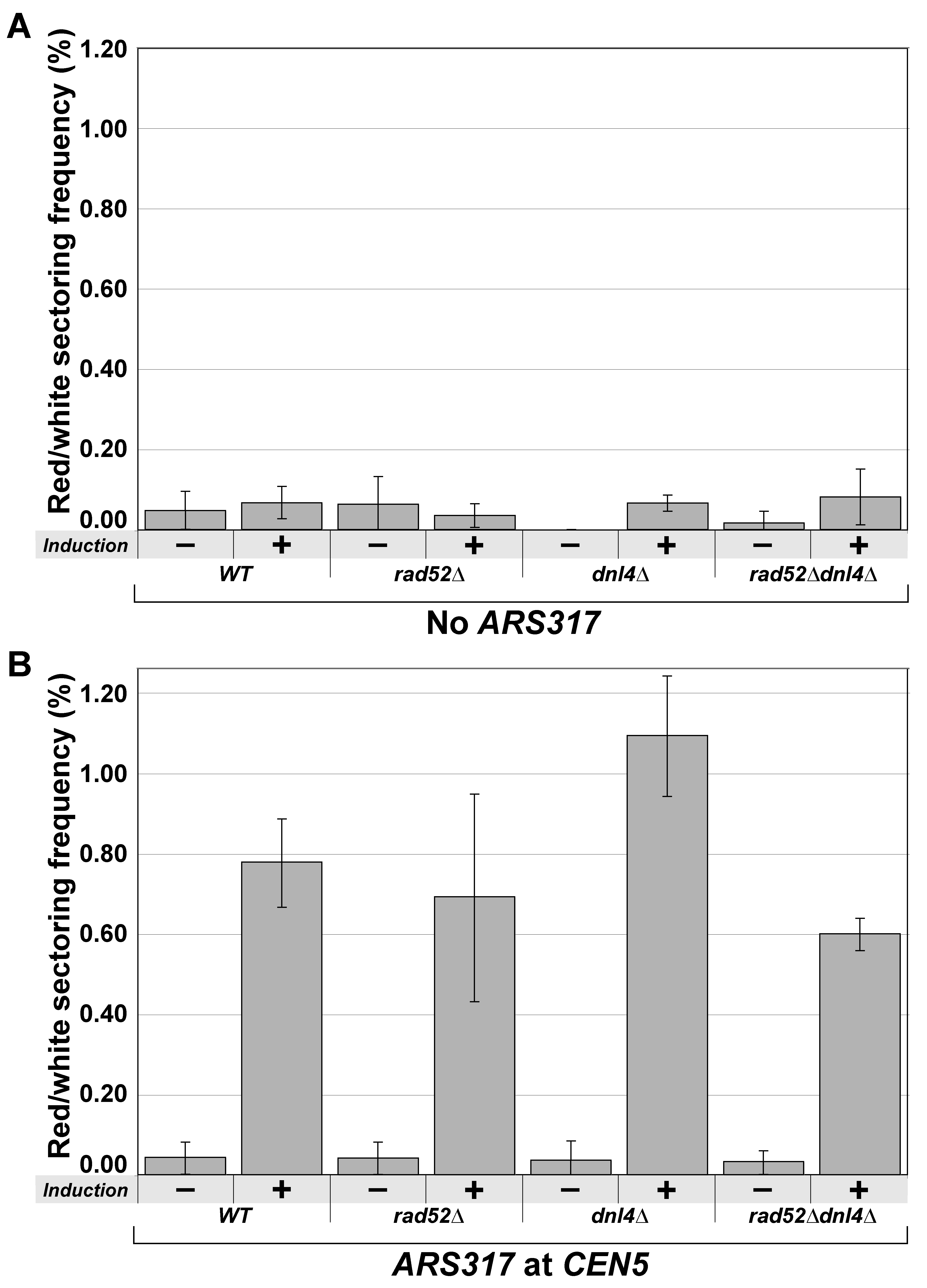

Supplement: S3 Fig — Diploid re-replicating strains with homozygous deletions of indicate genes and ade3–2p integrated at CEN5 were induced to re-replicate as described in Fig. 1A were scored for the frequency of red/white sectored colonies either before (-) or after (+) a 3 hr induction of re-replication (see S3 Table). Data is presented as the mean ± SD (n ≥ 3). (A) Strains containing no ARS317 (YJL9627). (B) Strains containing ARS317 integrated at CEN5 (YJL9637). (TIF) [file pgen.1005039.s003.tif]

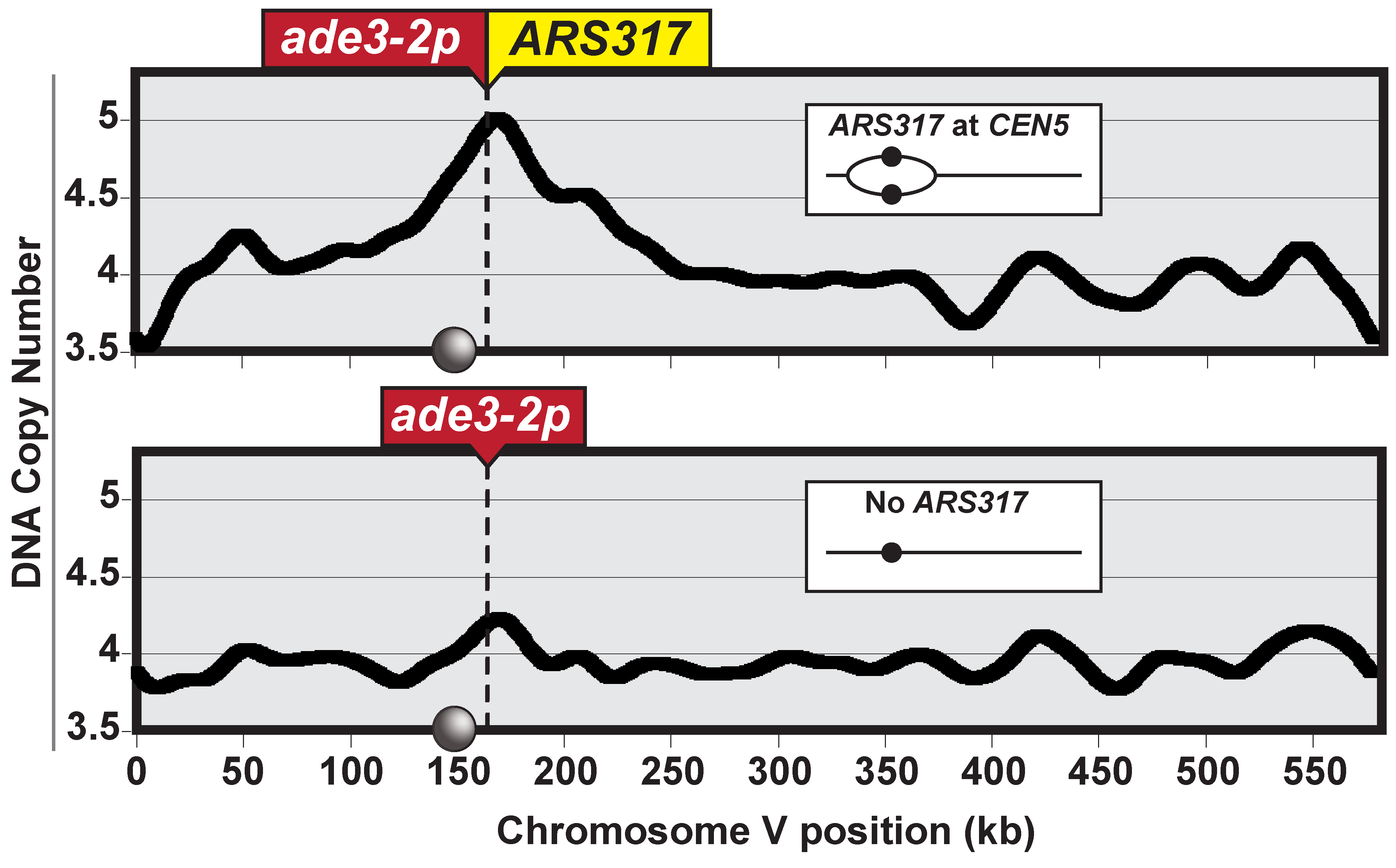

Supplement: S4 Fig — ARS317 and ade3–2p mark integration sites of the reinitiating origin and the copy number reporter, respectively. Inset shows schematic of re-replication bubbles inferred from profiles. Circles on X-axis and in schematic represent centromere CEN5. Upper panel: YJL9637. Lower panel: YJL9627. (TIF) [file pgen.1005039.s004.tif]

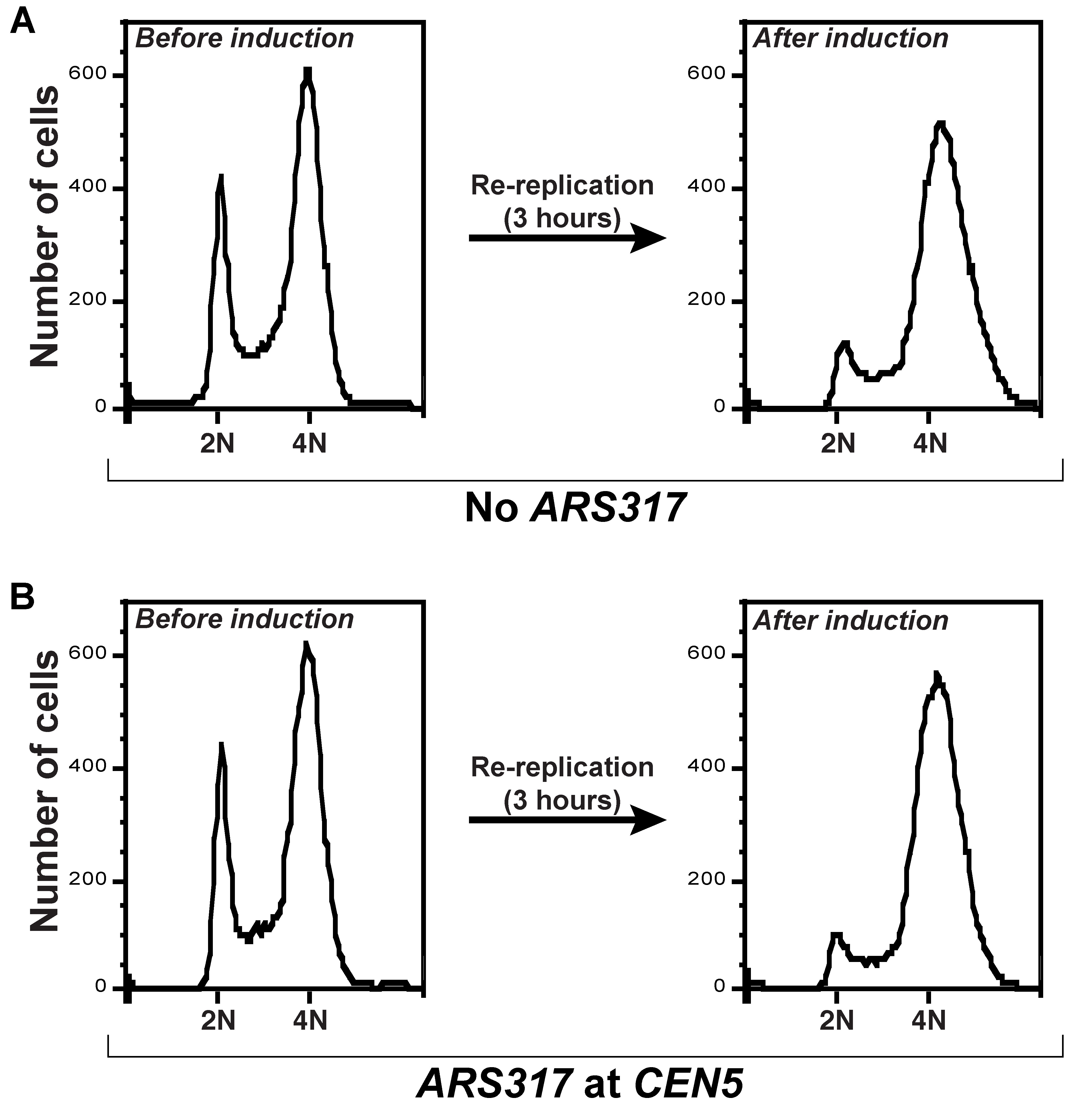

Supplement: S5 Fig — Flow cytometry of strains analyzed in S4 Fig before and after the induction of re-replication. (A) Strain containing no ARS317 (YJL9627). (B) Strain containing ARS317 integrated at CEN5 (YJL9637). (TIF) [file pgen.1005039.s005.tif]

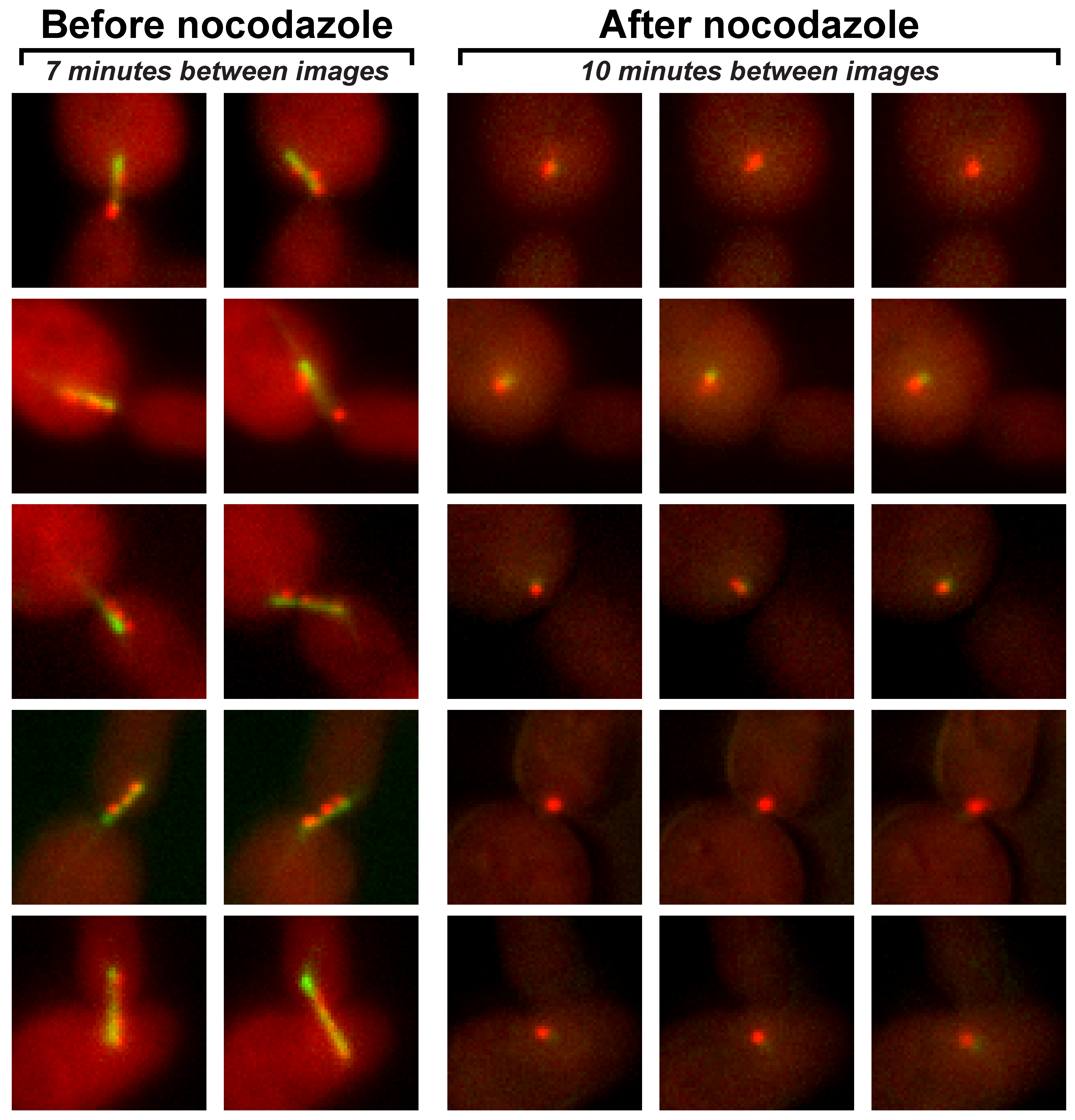

Supplement: S6 Fig — Centromeric re-replication was induced for 3 hr in asynchronously growing cells containing tdTomato marked centromeres and GFP tagged tubulin (YJL10671). Following the induction, which arrested cells in metaphase, time-lapse Z-stack images were taken every 7 min over approximately 20 min. Nocodazole was then added to eliminate the mitotic spindle, and imaging was continued every 10 min for 2 hr. Each row shows representative time-lapse images taken from a single cell either before nocodazole addition or after. (TIF) [file pgen.1005039.s006.tif]
