# Supplementary material for: Re-replication of a Centromere Induces Chromosomal Instability and Aneuploidy
Source: PLoS Genet. 2015 Apr 22;11(4):e1005039. doi: 10.1371/journal.pgen.1005039 (PMC4406714; doi:10.1371/journal.pgen.1005039)
Supplement: S1 Table — (DOCX) [file pgen.1005039.s007.docx]

**S1 Table. Array CGH for the re-replication profiles presented in this work.**

| **Strain** | **Relevant genotype** | **Figure** | **Sample no.  in GEO** |
| --- | --- | --- | --- |
| YJL9637 | ARS317 at CEN5 | 1B, top | GSM1340735 |
| YJL9629 | No ARS317 | 1B, middle | GSM1340736 |
| YJL9631 | ARS317 moved to Chr5_548 | 1B, bottom | GSM1340737 |
| YJL9637 | ARS317 at CEN5 | S2, top | GSM1346228 |
| YJL10171 | ARS317 at CEN5, *rad52∆* | S2, top middle | GSM1340738 |
| YJL10176 | ARS317 at CEN5, *dnl4∆* | S2, bottom middle | GSM1340739 |
| YJL10238 | ARS317 at CEN5, *rad52∆dnl4∆* | S2, bottom | GSM1340740 |
| YJL9637 | ARS317 at CEN5 (no arrest) | S4, top | GSM1340741 |
| YJL9627 | No ARS317 (no arrest) | S4, bottom | GSM1340742 |
