# Supplementary material for: Re-replication of a Centromere Induces Chromosomal Instability and Aneuploidy
Source: PLoS Genet. 2015 Apr 22;11(4):e1005039. doi: 10.1371/journal.pgen.1005039 (PMC4406714; doi:10.1371/journal.pgen.1005039)
Supplement: S2 Table — Chromosomes other than ChrV are at a copy number of 2.0 unless listed in “Other genomic changes” with copy number reported in parentheses. For chromosomal segments with a copy number other than 2.0, the boundaries of the segments are indicated by chromosomal coordinates within brackets. We inferred that the ade3–2p marked ChrV homolog had undergone a 2:0 segregation event if the total ChrV copy number was > 2.2 in the red sector and < 1.8 in the white sector (see Materials and Methods). LT = left telomere; RT = right telomere (DOCX) [file pgen.1005039.s008.docx]

| **Table S2: Array CGH results corresponding to Fig. 2.**  Chromosomes other than Chr5 are at a copy number of 2.0 unless listed in "Other genomic changes" with copy number reported in parentheses. For chromosomal segments with a copy number other than 2.0, the boundaries of the segments are indicated by chromosomal coordinates within brackets. We inferred that the ade3-2p marked Chr5 homolog had undergone a 2:0 segregation event if the total Chr5 copy number was > 2.2 in the red sector and < 1.8 in the white sector (see Materials and Methods). LT = left telomere; RT = right telomere. | | | | | | | |
| --- | --- | --- | --- | --- | --- | --- | --- |
| **Parental Strain** | **Relevant genotype** | **Colony number** | **Sector** | **Chr5 Copy No.** | **Other genomic changes** | **2:0 Chr5 segregation** | **Sample no.  in GEO** |
| YJL9627 | No ARS317 | SHE4-14-29R | Red | 2.6 | Chr10(2.7) | **+** | GSM1340743 |
| YJL9627 | No ARS317 | SHE4-14-29W | White | 1.5 | Chr10(1.5) |  | GSM1340744 |
| YJL9627 | No ARS317 | SHE4-14-30R | Red | 2.8 | – | **+** | GSM1340745 |
| YJL9627 | No ARS317 | SHE4-14-30W | White | 1.5 | Chr8(1.5) |  | GSM1340746 |
| YJL9627 | No ARS317 | SHE4-14-31R | Red | 2.8 | – | **+** | GSM1340747 |
| YJL9627 | No ARS317 | SHE4-14-31W | White | 1.2 | – |  | GSM1340748 |
| YJL9629 | No ARS317 | SHE5-29-9R | Red | 2.0 | Chr1(1) | **–** | GSM1340749 |
| YJL9629 | No ARS317 | SHE5-29-9W | White | 1.8 | Chr1(1.0); Chr9(2.2); Chr12{660kb-690kb(1.7)} |  | GSM1340750 |
| YJL9637 | ARS317 at CEN5 | SHE5-23-16R | Red | 2.7 | Chr12(2.3) | **+** | GSM1340751 |
| YJL9637 | ARS317 at CEN5 | SHE5-23-16W | White | 1.7 | Chr3{LT-5kb(2.5),6kb-90kb(3.6)}; Chr6{LT-140kb(1),140kb-RT(2.8)} |  | GSM1340752 |
| YJL9637 | ARS317 at CEN5 | SHE5-23-21R | Red | 2.0 | Chr8(1.2); Chr14(1.2) | **–** | GSM1340753 |
| YJL9637 | ARS317 at CEN5 | SHE5-23-21W | White | 1.5 | Chr8(1.2); Chr14(1.5) |  | GSM1340754 |
| YJL9637 | ARS317 at CEN5 | SHE5-23-22R | Red | 2.7 | *–* | **+** | GSM1340755 |
| YJL9637 | ARS317 at CEN5 | SHE5-23-22W | White | 1.3 | *–* |  | GSM1340756 |
| YJL9637 | ARS317 at CEN5 | SHE5-23-23R | Red | 2.6 | Chr16(2.7) | **+** | GSM1340757 |
| YJL9637 | ARS317 at CEN5 | SHE5-23-23W | White | 1.2 | *–* |  | GSM1340758 |
| YJL9637 | ARS317 at CEN5 | SHE4-14-65R | Red | 2.7 | – | **+** | GSM1340759 |
| YJL9637 | ARS317 at CEN5 | SHE4-14-65W | White | 1.5 | *–* |  | GSM1340760 |
| YJL9637 | ARS317 at CEN5 | SHE5-23-24R | Red | 2.8 | Chr10(2.75) | **+** | GSM1340761 |
| YJL9637 | ARS317 at CEN5 | SHE5-23-24W | White | 1.1 | Chr10(1.1) |  | GSM1340762 |
| YJL9639 | ARS317 at CEN5 | SHE5-25-16R | Red | 2.7 | Chr7{575kb-800kb(3)}; Chr12{950kb-975kb(3)} | **+** | GSM1340763 |
| YJL9639 | ARS317 at CEN5 | SHE5-25-16W | White | 1.3 | *–* |  | GSM1340764 |
| YJL9639 | ARS317 at CEN5 | SHE5-25-17R | Red | 2.0 | Chr3(2.7) | **–** | GSM1340765 |
| YJL9639 | ARS317 at CEN5 | SHE5-25-17W | White | 1.3 | – |  | GSM1340766 |
| YJL9639 | ARS317 at CEN5 | SHE5-25-19R | Red | 2.7 | – | **+** | GSM1340767 |
| YJL9639 | ARS317 at CEN5 | SHE5-25-19W | White | 1.6 | Chr16(2.7) |  | GSM1340768 |
| YJL9639 | ARS317 at CEN5 | SHE5-25-20R | Red | 2.7 | – | **+** | GSM1340769 |
| YJL9639 | ARS317 at CEN5 | SHE5-25-20W | White | 1.3 | *–* |  | GSM1340770 |
| YJL9639 | ARS317 at CEN5 | SHE5-25-23R | Red | 2.7 | Chr1(2.7) | **+** | GSM1340771 |
| YJL9639 | ARS317 at CEN5 | SHE5-25-23W | White | 1.4 | *–* |  | GSM1340772 |
