# Supplementary material for: Re-replication of a Centromere Induces Chromosomal Instability and Aneuploidy
Source: PLoS Genet. 2015 Apr 22;11(4):e1005039. doi: 10.1371/journal.pgen.1005039 (PMC4406714; doi:10.1371/journal.pgen.1005039)
Supplement: S3 Table — Chromosomes other than ChrV are at a copy number of 2.0 unless listed in “Other genomic changes” with copy number reported in parentheses. For chromosomal segments with a copy number other than 2.0, the boundaries of the segments are indicated by chromosomal coordinates within brackets. We inferred that the ade3–2p marked ChrV homolog had undergone a 2:1 segregation event if the total ChrV copy number was > 2.2 in the red sector and = 2.0 in the pink sector (see Materials and Methods). LT = left telomere; RT = right telomere; Mix = whole copy number of ChrV cannot be reported due to segmental gains or losses. (DOCX) [file pgen.1005039.s009.docx]

| **S3 Table. Array CGH results corresponding to Fig. 3.** Chromosomes other than Chr5 are at a copy number of 2.0 unless listed in "Other genomic changes" with copy number reported in parentheses. For chromosomal segments with a copy number other than 2.0, the boundaries of the segments are indicated by chromosomal coordinates within brackets. We inferred that the ade3-2p marked Chr5 homolog had undergone a 2:1 segregation event if the total Chr5 copy number was > 2.2 in the red sector and = 2.0 in the pink sector (see Materials and Methods). LT = left telomere; RT = right telomere; Mix = whole copy number of Chr5 cannot be reported due to segmental gains or losses. | | | | | | | |
| --- | --- | --- | --- | --- | --- | --- | --- |
| **Parental Strain** | **Relevant genotype** | **Colony number** | **Sector** | **Chr5 Copy No.** | **Other genomic changes** | **2:1 Chr5 segregation** | **Sample no.  in GEO** |
| YJL9627 | No ARS317 | SHE4-14-27R | Red | 2.0 | Chr7(2.7); Chr16(2.7) | **–** | GSM1340773 |
| YJL9627 | No ARS317 | SHE4-14-27P | Pink | 2.0 | Chr16(2.7) |  | GSM1340774 |
| YJL9627 | No ARS317 | SHE4-14-32R | Red | 2.0 | Chr2(2.7); Chr11{LT-370kb(1)}; Chr12{690kb-RT(2.8)} | **–** | GSM1340775 |
| YJL9627 | No ARS317 | SHE4-14-32P | Pink | 2.0 | Chr11(1.7) |  | GSM1340776 |
| YJL9627 | No ARS317 | SHE4-14-35R | Red | 2.7 | Chr13(2.7) | **+** | GSM1340777 |
| YJL9627 | No ARS317 | SHE4-14-35P | Pink | 2.0 | – |  | GSM1340778 |
| YJL9627 | No ARS317 | SHE4-14-36R | Red | Mix | Chr5{LT-160kb(2.9)}; Chr7{910kb-RT(2.9)} | **– *** | GSM1340779 |
| YJL9627 | No ARS317 | SHE4-14-36P | Pink | 2.0 | – |  | GSM1340780 |
| YJL9627 | No ARS317 | SHE4-14-38R | Red | 2.0 | Chr3(2.8) | **–** | GSM1340781 |
| YJL9627 | No ARS317 | SHE4-14-38P | Pink | 2.0 | Chr10(2.7) |  | GSM1340782 |
| YJL9627 | No ARS317 | SHE4-14-39R | Red | 2.7 | Chr3(2.5); Chr7{820kb-870kb(2.7)}; Chr11(2.7) | **+** | GSM1340783 |
| YJL9627 | No ARS317 | SHE4-14-39P | Pink | 2.0 | – |  | GSM1340784 |
| YJL9627 | No ARS317 | SHE4-14-40R | Red | 2.8 | Chr3{LT-170kb(2.6)}; Chr4{1200kb-RT(2.7)} | **+** | GSM1340785 |
| YJL9627 | No ARS317 | SHE4-14-40P | Pink | 2.0 | – |  | GSM1340786 |
| YJL9627 | No ARS317 | SHE4-14-41R | Red | Mix | Chr3(2.8); Chr5{450kb-RT(1)}; Chr15{LT-100kb(2.8)} | **–** | GSM1340787 |
| YJL9627 | No ARS317 | SHE4-14-41P | Pink | 2.0 | – |  | GSM1340788 |
| YJL9629 | No ARS317 | SHE5-29-8R | Red | 2.8 | Chr13(2.7) | **+** | GSM1340789 |
| YJL9629 | No ARS317 | SHE5-29-8P | Pink | 2.0 | – |  | GSM1340790 |
| YJL9629 | No ARS317 | SHE5-29-11R | Red | 2.0 | – | **–** | GSM1340791 |
| YJL9629 | No ARS317 | SHE5-29-11P | Pink | 2.0 | – |  | GSM1340792 |
| YJL9637 | ARS317 at CEN5 | SHE5-23-41R | Red | 2.7 | *–* | **+** | GSM1340793 |
| YJL9637 | ARS317 at CEN5 | SHE5-23-41P | Pink | 2.0 | Chr4{900kb-100kb(3)} |  | GSM1340794 |
| YJL9637 | ARS317 at CEN5 | SHE5-23-43R | Red | 2.8 | *–* | **+** | GSM1340795 |
| YJL9637 | ARS317 at CEN5 | SHE5-23-43P | Pink | 2.0 | – |  | GSM1340796 |
| YJL9637 | ARS317 at CEN5 | SHE4-14-88R | Red | 2.7 | – | **+** | GSM1340797 |
| YJL9637 | ARS317 at CEN5 | SHE4-14-88P | Pink | 2.0 | – |  | GSM1340798 |
| YJL9637 | ARS317 at CEN5 | SHE5-23-59R | Red | 2.7 | – | **+** | GSM1340799 |
| YJL9637 | ARS317 at CEN5 | SHE5-23-59P | Pink | 2.0 | *–* |  | GSM1340800 |
| YJL9637 | ARS317 at CEN5 | SHE5-23-64R | Red | 2.7 | Chr3{150kb-160kb(3)} | **+** | GSM1340801 |
| YJL9637 | ARS317 at CEN5 | SHE5-23-64P | Pink | 2.0 | Chr15(2.6) |  | GSM1340802 |
| YJL9637 | ARS317 at CEN5 | SHE5-23-53R | Red | Mix | Chr5{135kb-450kb(3), 450kb-RT(3.5)} | **– *** | GSM1340803 |
| YJL9637 | ARS317 at CEN5 | SHE5-23-53P | Pink | 2.0 | – |  | GSM1340804 |
| YJL9639 | ARS317 at CEN5 | SHE5-25-56R | Red | 2.7 | – | **+** | GSM1340805 |
| YJL9639 | ARS317 at CEN5 | SHE5-25-56P | Pink | 2.0 | – |  | GSM1340806 |
| YJL9639 | ARS317 at CEN5 | SHE5-25-58R | Red | 2.7 | Chr5{425kb-475kb(2)} | **+ **** | GSM1340807 |
| YJL9639 | ARS317 at CEN5 | SHE5-25-58P | Pink | 2.0 | Chr5{425kb-475kb(1)} |  | GSM1340808 |
| YJL9639 | ARS317 at CEN5 | SHE5-25-72R | Red | 2.6 | Chr12{650kb-700kb(1.1)} | **+** | GSM1340809 |
| YJL9639 | ARS317 at CEN5 | SHE5-25-72P | Pink | 2.0 | *–* |  | GSM1340810 |

**S3 Table (continued)**

| **Parental Strain** | **Relevant genotype** | **Colony number** | **Sector** | **Chr5 Copy No.** | **Other genomic changes** | **2:1 Chr5 segregation** | **Sample no.  in GEO** |
| --- | --- | --- | --- | --- | --- | --- | --- |
| YJL9639 | ARS317 at CEN5 | SHE5-25-80R | Red | 2.5 | – | **+** | GSM1340811 |
| YJL9639 | ARS317 at CEN5 | SHE5-25-80P | Pink | 2.0 | – |  | GSM1340812 |
| YJL9639 | ARS317 at CEN5 | SHE5-25-86R | Red | 2.7 | *–* | **– *** | GSM1340813 |
| YJL9639 | ARS317 at CEN5 | SHE5-25-86P | Pink | Mix | Chr5{LT-160kb(2.7), 540kb-RT(2.7)} |  | GSM1340814 |
| YJL10171 | ARS317 at CEN5, *rad52∆* | SHE5-01-93R | Red | 2.0 | Chr9(1.3); Chr14(1.9) | **–** | GSM1340815 |
| YJL10171 | ARS317 at CEN5, *rad52∆* | SHE5-01-93P | Pink | 2.0 | Chr1(1.2) |  | GSM1340816 |
| YJL10171 | ARS317 at CEN5, *rad52∆* | SHE5-01-99R | Red | 2.4 | – | **+** | GSM1340817 |
| YJL10171 | ARS317 at CEN5, *rad52∆* | SHE5-01-99P | Pink | 2.0 | – |  | GSM1340818 |
| YJL10171 | ARS317 at CEN5, *rad52∆* | SHE5-01-105R | Red | 2.7 | Chr1(1) | **+** | GSM1340819 |
| YJL10171 | ARS317 at CEN5, *rad52∆* | SHE5-01-105P | Pink | 2.0 | Chr1(1.2) |  | GSM1340820 |
| YJL10171 | ARS317 at CEN5, *rad52∆* | SHE5-01-109R | Red | 2.5 | – | **+** | GSM1340821 |
| YJL10171 | ARS317 at CEN5, *rad52∆* | SHE5-01-109P | Pink | 2.0 | – |  | GSM1340822 |
| YJL10171 | ARS317 at CEN5, *rad52∆* | SHE5-01-114R | Red | 2.5 | – | **+** | GSM1340823 |
| YJL10171 | ARS317 at CEN5, *rad52∆* | SHE5-01-114P | Pink | 2.0 | – |  | GSM1340824 |
| YJL10171 | ARS317 at CEN5, *rad52∆* | SHE5-01-118R | Red | 2.5 | Chr3(2.6) | **+** | GSM1340825 |
| YJL10171 | ARS317 at CEN5, *rad52∆* | SHE5-01-118P | Pink | 2.0 | Chr3(2.7) |  | GSM1340826 |
| YJL10171 | ARS317 at CEN5, *rad52∆* | SHE5-01-123R | Red | 2.9 | Chr14(1.1) | **+** | GSM1340827 |
| YJL10171 | ARS317 at CEN5, *rad52∆* | SHE5-01-123P | Pink | 2.0 | – |  | GSM1340828 |
| YJL10171 | ARS317 at CEN5, *rad52∆* | SHE5-01-129R | Red | 2.5 | Chr1(1); Chr16(2.5) | **+** | GSM1340829 |
| YJL10171 | ARS317 at CEN5, *rad52∆* | SHE5-01-129P | Pink | 2.0 | Chr1(1.1); Chr16(2.4) |  | GSM1340830 |
| YJL10171 | ARS317 at CEN5, *rad52∆* | SHE5-01-136R | Red | 2.5 | – | **+** | GSM1340831 |
| YJL10171 | ARS317 at CEN5, *rad52∆* | SHE5-01-136P | Pink | 2.0 | – |  | GSM1340832 |
| YJL10171 | ARS317 at CEN5, *rad52∆* | SHE5-01-141R | Red | 2.5 | Chr1(1.1) | **+** | GSM1340833 |
| YJL10171 | ARS317 at CEN5, *rad52∆* | SHE5-01-141P | Pink | 2.0 | – |  | GSM1340834 |
| YJL10176 | ARS317 at CEN5, *dnl4∆* | SHE5-01-149R | Red | 2.7 | – | **+** | GSM1340835 |
| YJL10176 | ARS317 at CEN5, *dnl4∆* | SHE5-01-149P | Pink | 2.0 | – |  | GSM1340836 |
| YJL10176 | ARS317 at CEN5, *dnl4∆* | SHE5-01-151R | Red | Mix | Chr5{160kb-540kb(2.6), 540kb-RT(2.7)} | **– *** | GSM1340837 |
| YJL10176 | ARS317 at CEN5, *dnl4∆* | SHE5-01-151P | Pink | 2.0 | – |  | GSM1340838 |
| YJL10176 | ARS317 at CEN5, *dnl4∆* | SHE5-01-155R | Red | 2.9 | – | **+** | GSM1340839 |
| YJL10176 | ARS317 at CEN5, *dnl4∆* | SHE5-01-155P | Pink | 2.0 | – |  | GSM1340840 |
| YJL10176 | ARS317 at CEN5, *dnl4∆* | SHE5-01-157R | Red | 2.8 | – | **+** | GSM1340841 |
| YJL10176 | ARS317 at CEN5, *dnl4∆* | SHE5-01-157P | Pink | 2.0 | Chr8(2.7); Chr9(2.7) |  | GSM1340842 |
| YJL10176 | ARS317 at CEN5, *dnl4∆* | SHE5-01-165R | Red | 2.8 | – | **+** | GSM1340843 |
| YJL10176 | ARS317 at CEN5, *dnl4∆* | SHE5-01-165P | Pink | 2.0 | – |  | GSM1340844 |
| YJL10176 | ARS317 at CEN5, *dnl4∆* | SHE5-01-171R | Red | 3.3 | Chr3(2.5); Chr9(2.5); Chr11(2.5); Chr16(2.5) | **+ ***** | GSM1340845 |
| YJL10176 | ARS317 at CEN5, *dnl4∆* | SHE5-01-171P | Pink | 2.5 | Chr3(2.5); Chr9(2.5); Chr11(3.3); Chr16(2.5) |  | GSM1340846 |
| YJL10176 | ARS317 at CEN5, *dnl4∆* | SHE5-01-173R | Red | 2.8 | – | **+** | GSM1340847 |
| YJL10176 | ARS317 at CEN5, *dnl4∆* | SHE5-01-173P | Pink | 2.0 | – |  | GSM1340848 |
| YJL10176 | ARS317 at CEN5, *dnl4∆* | SHE5-01-177R | Red | 2.9 | – | **+** | GSM1340849 |
| YJL10176 | ARS317 at CEN5, *dnl4∆* | SHE5-01-177P | Pink | 2.0 | – |  | GSM1340850 |

S3 Table (continued)

| **Parental Strain** | **Relevant genotype** | **Colony number** | **Sector** | **Chr5 Copy No.** | **Other genomic changes** | **2:1 Chr5 segregation** | | **Sample no.  in GEO** |
| --- | --- | --- | --- | --- | --- | --- | --- | --- |
| YJL10176 | ARS317 at CEN5, *dnl4∆* | SHE5-01-192R | Red | 2.7 | – | **+** | | GSM1340851 |
| YJL10176 | ARS317 at CEN5, *dnl4∆* | SHE5-01-192P | Pink | 2.0 | – |  |  | GSM1340852 |
| YJL10176 | ARS317 at CEN5, *dnl4∆* | SHE5-01-194R | Red | 2.8 | – | **+** | | GSM1340853 |
| YJL10176 | ARS317 at CEN5, *dnl4∆* | SHE5-01-194P | Pink | 2.0 | – |  |  | GSM1340854 |
| YJL10238 | ARS317 at CEN5, *rad52∆dnl4∆* | SHE5-23-85R | Red | 2.0 | Chr14(1.2) | **–** | | GSM1340855 |
| YJL10238 | ARS317 at CEN5, *rad52∆dnl4∆* | SHE5-23-85P | Pink | 2.0 | – |  |  | GSM1340856 |
| YJL10238 | ARS317 at CEN5, *rad52∆dnl4∆* | SHE5-23-87R | Red | 2.5 | Chr1(1) | **+** | | GSM1340857 |
| YJL10238 | ARS317 at CEN5, *rad52∆dnl4∆* | SHE5-23-87P | Pink | 2.0 | – |  |  | GSM1340858 |
| YJL10238 | ARS317 at CEN5, *rad52∆dnl4∆* | SHE5-23-89R | Red | 2.2 | – | **+** | | GSM1340859 |
| YJL10238 | ARS317 at CEN5, *rad52∆dnl4∆* | SHE5-23-89P | Pink | 2.0 | – |  |  | GSM1340860 |
| YJL10238 | ARS317 at CEN5, *rad52∆dnl4∆* | SHE5-23-91R | Red | 2.3 | – | **+** | | GSM1340861 |
| YJL10238 | ARS317 at CEN5, *rad52∆dnl4∆* | SHE5-23-91P | Pink | 2.0 | – |  |  | GSM1340862 |
| YJL10238 | ARS317 at CEN5, *rad52∆dnl4∆* | SHE5-23-93R | Red | 2.4 | Chr2(2.4) | **+** | | GSM1340863 |
| YJL10238 | ARS317 at CEN5, *rad52∆dnl4∆* | SHE5-23-93P | Pink | 2.0 | Chr1(1.6) |  |  | GSM1340864 |
| YJL10240 | ARS317 at CEN5, *rad52∆dnl4∆* | SHE5-25-119R | Red | 2.2 | – | **+** | | GSM1340865 |
| YJL10240 | ARS317 at CEN5, *rad52∆dnl4∆* | SHE5-25-119P | Pink | 2.0 | – |  |  | GSM1340866 |
| YJL10240 | ARS317 at CEN5, *rad52∆dnl4∆* | SHE5-25-129R | Red | 2.2 | – | **+** | | GSM1340867 |
| YJL10240 | ARS317 at CEN5, *rad52∆dnl4∆* | SHE5-25-129P | Pink | 2.0 | Chr1(2.4) |  |  | GSM1340868 |
| YJL10240 | ARS317 at CEN5, *rad52∆dnl4∆* | SHE5-25-130R | Red | 2.2 | – | **+** | | GSM1340869 |
| YJL10240 | ARS317 at CEN5, *rad52∆dnl4∆* | SHE5-25-130P | Pink | 2.0 | – |  |  | GSM1340870 |
| YJL10240 | ARS317 at CEN5, *rad52∆dnl4∆* | SHE5-25-134R | Red | 2.4 | – | **+** | | GSM1340871 |
| YJL10240 | ARS317 at CEN5, *rad52∆dnl4∆* | SHE5-25-134P | Pink | 2.0 | – |  |  | GSM1340872 |
| YJL10240 | ARS317 at CEN5, *rad52∆dnl4∆* | SHE5-25-135R | Red | 2.4 | – | **+** | | GSM1340873 |
| YJL10240 | ARS317 at CEN5, *rad52∆dnl4∆* | SHE5-25-135P | Pink | 2.0 | – |  |  | GSM1340874 |
|  |  |  |  |  |  |  |  |  |
| * Segment of Chr5 containing ade3-2p was at higher copy number than 2.0, accounting for red color of sector. However, the entire chromosome was not affected, so these isolates were not scored as 2:1 segregation events. | | | | | | | | |
| ** Small segemental copy number loss in Chr5 in both sectors was consistent with a pre-exisiting deletion in one of the Chr5 homologs. Remaining copy number data was consistent with a 2:1 segregation event and colony was scored as such. | | | | | | | | |
| *** Copy number of Chr5 in both sectors and sector color was consistent with a pre-existing extra copy of the non-rereplicating Chr5 homolog. Remaining copy number data was consistent with a 2:1 segregation event and colony was scored as such. | | | | | | | | |
