# Supplementary material for: Re-replication of a Centromere Induces Chromosomal Instability and Aneuploidy
Source: PLoS Genet. 2015 Apr 22;11(4):e1005039. doi: 10.1371/journal.pgen.1005039 (PMC4406714; doi:10.1371/journal.pgen.1005039)
Supplement: S4 Table — Chromosomes other than ChrV are at a copy number of 2.0 unless listed in “Other genomic changes” with copy number reported in parentheses. For chromosomal segments with a copy number other than 2.0, the boundaries of the segments are indicated by chromosomal coordinates within brackets. We inferred that the ade3–2p marked ChrV homolog had undergone a 2:0 segregation event if the total ChrV copy number was > 2.2 in the red sector and < 1.8 in the white sector (see Materials and Methods). LT = left telomere; RT = right telomere; Mix = whole copy number of ChrV cannot be reported due to segmental gains or losses. (DOCX) [file pgen.1005039.s010.docx]

| **S4 Table. Array CGH results corresponding to Fig. 4B.** Chromosomes other than Chr5 are at a copy number of 2.0 unless listed in "Other genomic changes" with copy number reported in parentheses. For chromosomal segments with a copy number other than 2.0, the boundaries of the segments are indicated by chromosomal coordinates within brackets. We inferred that the ade3-2p marked Chr5 homolog had undergone a 2:0 segregation event if the total Chr5 copy number was > 2.2 in the red sector and < 1.8 in the white sector (see Materials and Methods). LT = left telomere; RT = right telomere; Mix = whole copy number of Chr5 cannot be reported due to segmental gains or losses. | | | | | | | | |
| --- | --- | --- | --- | --- | --- | --- | --- | --- |
| **Parental Strain** | **Relevant genotype** | **Colony number** | **Sector** | **Chr5 Copy No.** | **Other genomic changes** | **2:0 Chr5 segregation** | | **Sample no.  in GEO** |
| YJL9627 | No ARS317 (no arrest) | SHE5-24-20R | Red | 2.8 | – | **+** | | GSM1340875 |
| YJL9627 | No ARS317 (no arrest) | SHE5-24-20W | White | 1.8 | – |  |  | GSM1340876 |
| YJL9637 | ARS317 at CEN5 (no arrest) | SHE5-24-12R | Red | 2.7 | – | **+** | | GSM1340877 |
| YJL9637 | ARS317 at CEN5 (no arrest) | SHE5-24-12W | White | 1.7 | – |  |  | GSM1340878 |
| YJL9637 | ARS317 at CEN5 (no arrest) | SHE5-24-14R | Red | 2.6 | Chr3(2.7); Chr12(2.6) | **+** | | GSM1340879 |
| YJL9637 | ARS317 at CEN5 (no arrest) | SHE5-24-14W | White | 1.3 | Chr3(2.9) |  |  | GSM1340880 |
| YJL9637 | ARS317 at CEN5 (no arrest) | SHE5-24-15-R | Red | 2.8 | – | **+** | | GSM1340881 |
| YJL9637 | ARS317 at CEN5 (no arrest) | SHE5-24-15W | White | 1.7 | Chr1(2.7) |  |  | GSM1340882 |
| YJL9637 | ARS317 at CEN5 (no arrest) | SHE5-24-16R | Red | 2.7 | – | **+** | | GSM1340883 |
| YJL9637 | ARS317 at CEN5 (no arrest) | SHE5-24-16W | White | 1.2 | Chr7{810kb-880kb(3)}; Chr12{970kb-RT(1.1)}; Chr15{RT-120kb(3)} |  |  | GSM1340884 |
| YJL9637 | ARS317 at CEN5 (no arrest) | SHE5-24-18R | Red | 2.8 | – | **+** | | GSM1340885 |
| YJL9637 | ARS317 at CEN5 (no arrest) | SHE5-24-18W | White | 1.2 | Chr1(1.1) |  |  | GSM1340886 |
| YJL9637 | ARS317 at CEN5 (no arrest) | SHE5-24-19R | Red | Mix | Chr5{LT-140kb(4), 140kb-290kb(3.2), 290kb-RT(2.5)}; Chr7 (2.5); Chr11(2.5); Chr16(2.5) | **– *** | | GSM1340887 |
| YJL9637 | ARS317 at CEN5 (no arrest) | SHE5-24-19W | White | 1.3 | – |  |  | GSM1340888 |
| YJL9639 | ARS317 at CEN5 (no arrest) | SHE5-24-21R | Red | Mix | Chr2{260kb-RT(2.7)}; Chr3{LT-5kb(2.5), 10kb-90kb(3.3), 90kb-290kb(2.5)}; Chr5{LT-150kb(3), 150kb-RT(2.7)} | **– *** | | GSM1340889 |
| YJL9639 | ARS317 at CEN5 (no arrest) | SHE5-24-21W | White | 1.5 | – |  |  | GSM1340890 |
| YJL9639 | ARS317 at CEN5 (no arrest) | SHE5-24-25R | Red | 2.7 | Chr8(2.7) | **+** | | GSM1340891 |
| YJL9639 | ARS317 at CEN5 (no arrest) | SHE5-24-25W | White | 1.2 | Chr12{660kb-690kb(1.2)} |  |  | GSM1340892 |
| YJL9639 | ARS317 at CEN5 (no arrest) | SHE5-24-28R | Red | 2.8 | – | **+** | | GSM1340893 |
| YJL9639 | ARS317 at CEN5 (no arrest) | SHE5-24-28W | White | 1.3 | – |  |  | GSM1340894 |
| YJL9639 | ARS317 at CEN5 (no arrest) | SHE5-24-29R | Red | 2.8 | – | **+** | | GSM1340895 |
| YJL9639 | ARS317 at CEN5 (no arrest) | SHE5-24-29W | White | 1.2 | – |  |  | GSM1340896 |
|  |  |  |  |  |  |  |  |  |
| * Copy number of Chr5 in both sectors was consistent with 2:0 segregation of Chr5. However, because of additional segmental copy number increases affecting Chr5 in the red sector, other explanations besides whole-chromosome missegregation were possible, hence these colonies were not scored as 2:0 segregation events. | | | | | | | | |
