# Supplementary material for: Re-replication of a Centromere Induces Chromosomal Instability and Aneuploidy
Source: PLoS Genet. 2015 Apr 22;11(4):e1005039. doi: 10.1371/journal.pgen.1005039 (PMC4406714; doi:10.1371/journal.pgen.1005039)
Supplement: S5 Table — Chromosomes other than ChrV are at a copy number of 2.0 unless listed in “Other genomic changes” with copy number reported in parentheses. For chromosomal segments with a copy number other than 2.0, the boundaries of the segments are indicated by chromosomal coordinates within brackets. We inferred that the ade3–2p marked ChrV homolog had undergone a 2:1 segregation event if the total ChrV copy number was > 2.2 in the red sector and = 2.0 in the pink sector (see Materials and Methods). LT = left telomere; RT = right telomere; Mix = whole copy number of ChrV cannot be reported due to segmental gains or losses. (DOCX) [file pgen.1005039.s011.docx]

| **S5 Table. Array CGH results corresponding to Fig. 4D.**  Chromosomes other than Chr5 are at a copy number of 2.0 unless listed in "Other genomic changes" with copy number reported in parentheses. For chromosomal segments with a copy number other than 2.0, the boundaries of the segments are indicated by chromosomal coordinates within brackets. We inferred that the *ade3-2p* marked Chr5 homolog had undergone a 2:1 segregation event if the total Chr5 copy number was > 2.2 in the red sector and = 2.0 in the pink sector (see Materials and Methods). LT = left telomere; RT = right telomere; Mix = whole copy number of Chr5 cannot be reported due to segmental gains or losses. | | | | | | | |
| --- | --- | --- | --- | --- | --- | --- | --- |
| **Parental Strain** | **Relevant genotype** | **Colony number** | **Sector** | **Chr5 Copy No.** | **Other genomic changes** | **2:1 Chr5 segregation** | **Sample no.  in GEO** |
| YJL9627 | No ARS317 (no arrest) | SHE5-24-1R | Red | 2.0 | Chr3{LT-5kb(2.7), 6kb-90kb(3.5), 90kb-RT(2.7)}; Chr12{975kb-RT(1.1)}; Chr13(2.7) | **–** | GSM1340897 |
| YJL9627 | No ARS317 (no arrest) | SHE5-24-1P | Pink | 2.0 | – |  | GSM1340898 |
| YJL9627 | No ARS317 (no arrest) | SHE5-24-2R | Red | 2.7 | – | **+** | GSM1340899 |
| YJL9627 | No ARS317 (no arrest) | SHE5-24-2P | Pink | 2.0 | Chr4{900kb-980kb(3)} |  | GSM1340900 |
| YJL9627 | No ARS317 (no arrest) | SHE5-24-3R | Red | 2.0 | Chr3(2.7) | **–** | GSM1340901 |
| YJL9627 | No ARS317 (no arrest) | SHE5-24-3P | Pink | 2.0 | Chr1(2.7) |  | GSM1340902 |
| YJL9627 | No ARS317 (no arrest) | SHE5-24-4R | Red | 2.7 | – | **+** | GSM1340903 |
| YJL9627 | No ARS317 (no arrest) | SHE5-24-4P | Pink | 2.0 | – |  | GSM1340904 |
| YJL9627 | No ARS317 (no arrest) | SHE5-24-5R | Red | Mix | Chr4{1000kb-RT(2.7)}; Chr5{LT-440kb(2.7)} | **– *** | GSM1340905 |
| YJL9627 | No ARS317 (no arrest) | SHE5-24-5P | Pink | 2.0 | – |  | GSM1340906 |
| YJL9627 | No ARS317 (no arrest) | SHE5-24-6R | Red | 2.7 | Chr1(2.6) | **+** | GSM1340907 |
| YJL9627 | No ARS317 (no arrest) | SHE5-24-6P | Pink | 2.0 | Chr1(2.6) |  | GSM1340908 |
| YJL9627 | No ARS317 (no arrest) | SHE5-24-7R | Red | 2.7 | Chr3{150kb-170kb(2.6)} | **+** | GSM1340909 |
| YJL9627 | No ARS317 (no arrest) | SHE5-24-7P | Pink | 2.0 | Chr3{150kb-170kb(2.6)}; Chr8{LT-200kb(2.7)}; Chr15{140kb-RT(2.7)} |  | GSM1340910 |
| YJL9627 | No ARS317 (no arrest) | SHE5-24-8R | Red | 2.7 | – | **+** | GSM1340911 |
| YJL9627 | No ARS317 (no arrest) | SHE5-24-8P | Pink | 2.0 | – |  | GSM1340912 |
| YJL9627 | No ARS317 (no arrest) | SHE5-24-9R | Red | 2.7 | – | **+** | GSM1340913 |
| YJL9627 | No ARS317 (no arrest) | SHE5-24-9P | Pink | 2.0 | – |  | GSM1340914 |
| YJL9627 | No ARS317 (no arrest) | SHE5-24-10R | Red | 2.6 | Chr6(2.6); Chr8(2.6) | **+** | GSM1340915 |
| YJL9627 | No ARS317 (no arrest) | SHE5-24-10P | Pink | 2.0 | Chr1(3.5); Chr3(2.6); Chr13(2.6) |  | GSM1340916 |
| YJL9637 | ARS317 at CEN5 (no arrest) | SHE5-24-32R | Red | 2.7 | Chr1(2.7) | **+** | GSM1340917 |
| YJL9637 | ARS317 at CEN5 (no arrest) | SHE5-24-32P | Pink | 2.0 | Chr10(2.7) |  | GSM1340918 |
| YJL9637 | ARS317 at CEN5 (no arrest) | SHE5-24-35R | Red | 2.7 | – | **+** | GSM1340919 |
| YJL9637 | ARS317 at CEN5 (no arrest) | SHE5-24-35P | Pink | 2.0 | – |  | GSM1340920 |
| YJL9637 | ARS317 at CEN5 (no arrest) | SHE5-24-39R | Red | 3.4 | Chr3(2.5); Chr10(2.7) | **+** | GSM1340921 |
| YJL9637 | ARS317 at CEN5 (no arrest) | SHE5-24-39P | Pink | 2.0 | Chr2(2.7) |  | GSM1340922 |
| YJL9637 | ARS317 at CEN5 (no arrest) | SHE5-24-44R | Red | 2.8 | – | **+** | GSM1340923 |
| YJL9637 | ARS317 at CEN5 (no arrest) | SHE5-24-44P | Pink | 2.0 | – |  | GSM1340924 |
| YJL9637 | ARS317 at CEN5 (no arrest) | SHE5-24-47R | Red | 2.7 | Chr4{880kb-980kb(3)}; Chr7{575kb-RT(3)}; Chr16{LT-70kb(1)} | **+** | GSM1340925 |
| YJL9637 | ARS317 at CEN5 (no arrest) | SHE5-24-47P | Pink | 2.0 | Chr4{880kb-980kb(3)}; Chr7{575kb-RT(3)}; Chr16{LT-70kb(1)} |  | GSM1340926 |
| YJL9637 | ARS317 at CEN5 (no arrest) | SHE5-24-52R | Red | 2.7 | – | **+** | GSM1340927 |
| YJL9637 | ARS317 at CEN5 (no arrest) | SHE5-24-52P | Pink | 2.0 | – |  | GSM1340928 |
| YJL9639 | ARS317 at CEN5 (no arrest) | SHE5-24-63R | Red | 2.7 | – | **+** | GSM1340929 |
| YJL9639 | ARS317 at CEN5 (no arrest) | SHE5-24-63P | Pink | 2.0 | – |  | GSM1340930 |
| YJL9639 | ARS317 at CEN5 (no arrest) | SHE5-24-68R | Red | 2.7 | – | **+** | GSM1340931 |
| YJL9639 | ARS317 at CEN5 (no arrest) | SHE5-24-68P | Pink | 2.0 | – |  | GSM1340932 |

**S5 Table (continued)**

| **Parental Strain** | **Relevant genotype** | **Colony number** | **Sector** | **Chr5 Copy No.** | **Other genomic changes** | **2:1 Chr5 segregation** | **Sample no.  in GEO** |
| --- | --- | --- | --- | --- | --- | --- | --- |
| YJL9639 | ARS317 at CEN5 (no arrest) | SHE5-24-74R | Red | 2.8 | – | **+** | GSM1340933 |
| YJL9639 | ARS317 at CEN5 (no arrest) | SHE5-24-74P | Pink | 2.0 | Chr3{155kb-170kb(2.7)} |  | GSM1340934 |
| YJL9639 | ARS317 at CEN5 (no arrest) | SHE5-24-76R | Red | 2.8 | – | **+** | GSM1340935 |
| YJL9639 | ARS317 at CEN5 (no arrest) | SHE5-24-76P | Pink | 2.0 | – |  | GSM1340936 |
| * Segment of Chr5 containing ade3-2p was at higher copy number than 2.0, accounting for red color of sector. However, the entire chromosome was not affected, so these isolates were not scored as 2:1 segregation events. | | | | | | | |
