# Supplementary material for: Re-replication of a Centromere Induces Chromosomal Instability and Aneuploidy
Source: PLoS Genet. 2015 Apr 22;11(4):e1005039. doi: 10.1371/journal.pgen.1005039 (PMC4406714; doi:10.1371/journal.pgen.1005039)
Supplement: S6 Table — After induction of re-replication, the number of resolvable spots in each cell was counted before and after nocodazole treatment (see Materials and Methods). Cells with the same number of spots before nocodazole treatment were grouped together and each group was then categorized based on the number of spots that could still be resolved after nocodazole treatment. Percentages are based on the total number of cells scored for the indicated initial spot number. (DOCX) [file pgen.1005039.s012.docx]

**S6 Table. Scoring fluorescent spots marking *CEN5* before and after nocodazole treatment.** After induction of re-replication, the number of resolvable spots in each cell was counted before and after nocodazole treatment (see Materials and Methods). Cells with the same number of spots before nocodazole treatment were grouped together and each group was then categorized based on the number of spots that could still be resolved after nocodazole treatment. Percentages are based on the total number of cells scored for the indicated initial spot number.

|  | **No *ARS317*** (Trial 1: 111 cells, Trial 2:100 cells) | | | | | | ***ARS317* at *CEN5*** (Trial 1: 109 cells, Trial 2:114 cells) | | | | | |
| --- | --- | --- | --- | --- | --- | --- | --- | --- | --- | --- | --- | --- |
|  | Initially 2 spots | | Initially 3 spots | | Initially 4 spots | | Initially 2 spots | | Initially 3 spots | | Initially 4 spots | |
|  | *Trial 1* | *Trial 2* | *Trial 1* | *Trial 2* | *Trial 1* | *Trial 2* | *Trial 1* | *Trial 2* | *Trial 1* | *Trial 2* | *Trial 1* | *Trial 2* |
| 1 spot after nocodazole | **92** (83%) | **87** (87%) | 0 | 0 | 0 | 0 | **36**  (77%) | **31**  (72%) | **29**  (56%) | **36**  (69%) | **3**  (30%) | **4**  (21%) |
| 2 spots after nocodazole | **19** (17%) | **13** (13%) | 0 | 0 | 0 | 0 | **11**  (23%) | **12**  (28%) | **19**  (37%) | **11**  (21%) | **4**  (40%) | **6**  (32%) |
| 3 spots after nocodazole | 0 | 0 | 0 | 0 | 0 | 0 | 0 | 0 | **4**  (8%) | **5**  (10%) | **2**  (20%) | **6**  (32%) |
| 4 spots after nocodazole | 0 | 0 | 0 | 0 | 0 | 0 | 0 | 0 | 0 | 0 | **1**  (10%) | **3**  (16%) |
| Totals | 111 | 100 | 0 | 0 | 0 | 0 | 47 | 43 | 52 | 52 | 10 | 19 |
|  |  |  |  |  |  |  |  |  |  |  |  |  |
|  |  | | |  | | |  | | |  | | |
