# Supplementary material for: Re-replication of a Centromere Induces Chromosomal Instability and Aneuploidy
Source: PLoS Genet. 2015 Apr 22;11(4):e1005039. doi: 10.1371/journal.pgen.1005039 (PMC4406714; doi:10.1371/journal.pgen.1005039)
Supplement: S7 Table — Colony counts and sector frequencies are shown for each experimental trial. Mean frequencies (not weighted for trial size), standard deviations, and p-values from t-test statistical analyses (see Materials and Methods) were calculated from these sector frequencies. (DOCX) [file pgen.1005039.s013.docx]

| **S7 Table. Results of all re-replication assays reported in this work.** Colony counts and sector frequencies are shown for each experimental trial. Mean frequencies (not weighted for trial size), standard deviations, and *p*-values from *t*-test statistical analyses (see Materials and Methods) were calculated from these sector frequencies. | | | | | | | | | | | | | |
| --- | --- | --- | --- | --- | --- | --- | --- | --- | --- | --- | --- | --- | --- |
| **Relevant genotype** | **Strain** | **Trial No.** | **Sectoring Frequencies** | | | |  | **Actual Colony Counts** | | | | | |
|  |  |  | **Before induction** | | **After Induction** | |  | **Before Induction** | | | **After Induction** | | |
|  |  |  | **Red/White** | **Red/Pink** | **Red/White** | **Red/Pink** |  | **Red/White** | **Red/Pink** | **All colonies** | **Red/White** | **Red/Pink** | **All colonies** |
| ARS317 at CEN5 | 9637 | 1 | 0.00% | 0.24% | 0.85% | 2.32% |  | 0 | 4 | 1655 | 34 | 93 | 4014 |
|  | 9637 | 2 | 0.06% | 0.25% | 0.88% | 2.22% |  | 2 | 8 | 3141 | 40 | 101 | 4545 |
|  | 9639 | 3 | 0.07% | 0.22% | 0.80% | 2.57% |  | 2 | 6 | 2767 | 29 | 93 | 3620 |
|  | 9637 | 4 | 0.10% | 0.10% | 0.78% | 2.57% |  | 2 | 2 | 2044 | 14 | 46 | 1787 |
|  | 9637 | 5 | 0.00% | 0.14% | 0.83% | 2.99% |  | 0 | 3 | 2083 | 16 | 58 | 1938 |
|  | 9639 | 6 | 0.05% | 0.16% | 0.79% | 2.06% |  | 1 | 3 | 1918 | 16 | 42 | 2035 |
|  | 9637 | 7 | 0.10% | 0.25% | 0.71% | 2.07% |  | 2 | 5 | 2000 | 11 | 32 | 1545 |
|  | 9639 | 8 | 0.00% | 0.15% | 0.93% | 2.94% |  | 0 | 3 | 1991 | 17 | 54 | 1837 |
|  | 9637 | 9 | 0.00% | 0.21% | 0.67% | 2.06% |  | 0 | 4 | 1923 | 12 | 37 | 1798 |
|  | 9637 | 10 | 0.10% | 0.10% | 0.54% | 1.94% |  | 2 | 2 | 2082 | 10 | 36 | 1853 |
|  | 9639 | 11 | 0.00% | 0.19% | 0.81% | 2.89% |  | 0 | 3 | 1592 | 17 | 61 | 2110 |
|  | *Mean of trials* | | 0.04% | 0.18% | 0.78% | 2.42% | *Totals* | 11 | 43 | 23196 | 216 | 653 | 27082 |
|  | *SD (sample)* | | 0.04% | 0.06% | 0.11% | 0.39% |  |  |  |  |  |  |  |
|  |  |  |  |  |  |  |  |  |  |  |  |  |  |
| No ARS317 | 9627 | 1 | 0.05% | 0.38% | 0.10% | 0.27% |  | 1 | 7 | 1819 | 4 | 11 | 4083 |
|  | 9627 | 2 | 0.00% | 0.20% | 0.08% | 0.25% |  | 0 | 6 | 3061 | 4 | 12 | 4756 |
|  | 9629 | 3 | 0.09% | 0.00% | 0.02% | 0.16% |  | 1 | 0 | 1155 | 1 | 8 | 4893 |
|  | *Mean of trials* | | 0.05% | 0.19% | 0.07% | 0.23% | *Totals* | 2 | 13 | 6035 | 9 | 31 | 13732 |
|  | *SD (sample)* | | 0.05% | 0.19% | 0.04% | 0.06% |  |  |  |  |  |  |  |
|  |  |  |  |  |  |  |  |  |  |  |  |  |  |
|  |  |  |  |  |  |  |  |  |  |  |  |  |  |
| ARS317 moved to Chr5_548 | 9631 | 1 | 0.14% | 0.14% | 0.08% | 0.21% |  | 2 | 2 | 1451 | 3 | 8 | 3878 |
|  | 9631 | 2 | 0.08% | 0.21% | 0.02% | 0.40% |  | 2 | 5 | 2399 | 1 | 19 | 4794 |
|  | 9633 | 3 | 0.00% | 0.13% | 0.14% | 0.14% |  | 0 | 3 | 2274 | 5 | 5 | 3461 |
|  | *Mean of trials* | | 0.07% | 0.16% | 0.08% | 0.25% | *Totals* | 4 | 10 | 6124 | 9 | 32 | 12133 |
|  | *SD (sample)* | | 0.07% | 0.04% | 0.06% | 0.13% |  |  |  |  |  |  |  |
|  |  |  |  |  |  |  |  |  |  |  |  |  |  |

S7 Table (continued)

| **Relevant genotype** | **Strain** | **Trial No.** | **Sectoring Frequencies** | | | |  | **Actual Colony Counts** | | | | | |
| --- | --- | --- | --- | --- | --- | --- | --- | --- | --- | --- | --- | --- | --- |
|  |  |  | **Before induction** | | **After Induction** | |  | **Before Induction** | | | **After Induction** | | |
|  |  |  | **Red/White** | **Red/Pink** | **Red/White** | **Red/Pink** |  | **Red/White** | **Red/Pink** | **All colonies** | **Red/White** | **Red/Pink** | **All colonies** |
| ARS317 at CEN5, ∆rad52 | 10171 | 1 | 0.05% | 0.15% | 0.96% | 1.26% |  | 1 | 3 | 1995 | 19 | 25 | 1978 |
|  | 10171 | 2 | 0.06% | 0.22% | 0.52% | 1.13% |  | 1 | 4 | 1810 | 10 | 22 | 1939 |
|  | 10172 | 3 | 0.00% | 0.14% | 0.54% | 0.98% |  | 0 | 3 | 2145 | 11 | 20 | 2049 |
|  | 10171 | 4 | 0.11% | 0.26% | 0.98% | 1.09% |  | 2 | 5 | 1901 | 18 | 20 | 1833 |
|  | 10172 | 5 | 0.00% | 0.50% | 0.47% | 1.25% |  | 0 | 10 | 1995 | 9 | 24 | 1913 |
|  | *Mean of trials* | | 0.04% | 0.26% | 0.69% | 1.14% | *Totals* | 4 | 25 | 9846 | 67 | 111 | 9712 |
|  | *SD (sample)* | | 0.04% | 0.15% | 0.26% | 0.12% |  |  |  |  |  |  |  |
|  |  |  |  |  |  |  |  |  |  |  |  |  |  |
| ARS317 at CEN5, ∆dnl4 | 10176 | 1 | 0.00% | 0.48% | 1.15% | 7.05% |  | 0 | 0 | 2097 | 27 | 165 | 2339 |
|  | 10176 | 2 | 0.05% | 0.29% | 1.19% | 8.77% |  | 1 | 6 | 2068 | 13 | 96 | 1095 |
|  | 10177 | 3 | 0.10% | 0.29% | 1.17% | 7.40% |  | 2 | 6 | 2070 | 12 | 76 | 1027 |
|  | 10177 | 4 | 0.00% | 0.31% | 0.87% | 6.40% |  | 0 | 7 | 2267 | 9 | 66 | 1031 |
|  | *Mean of trials* | | 0.04% | 0.34% | 1.10% | 7.41% | *Totals* | 3 | 19 | 8502 | 61 | 403 | 5492 |
|  | *SD (sample)* | | 0.05% | 0.09% | 0.15% | 1.00% |  |  |  |  |  |  |  |
|  |  |  |  |  |  |  |  |  |  |  |  |  |  |
|  |  |  |  |  |  |  |  |  |  |  |  |  |  |
| No ARS317, ∆rad52 | 10164 | 1 | 0.05% | 0.38% | 0.06% | 0.79% |  | 1 | 8 | 2120 | 1 | 14 | 1772 |
|  | 10165 | 2 | 0.00% | 0.56% | 0.05% | 1.03% |  | 0 | 11 | 1981 | 1 | 21 | 2040 |
|  | 10164 | 3 | 0.14% | 0.42% | 0.00% | 0.94% |  | 3 | 9 | 2133 | 0 | 22 | 2346 |
|  | *Mean of trials* | | 0.06% | 0.45% | 0.04% | 0.92% | *Totals* | 4 | 28 | 6234 | 2 | 57 | 6158 |
|  | *SD (sample)* | | 0.07% | 0.09% | 0.03% | 0.12% |  |  |  |  |  |  |  |
|  |  |  |  |  |  |  |  |  |  |  |  |  |  |
|  |  |  |  |  |  |  |  |  |  |  |  |  |  |
| No ARS317, ∆dnl4 | 10168 | 1 | 0.00% | 0.19% | 0.05% | 0.66% |  | 0 | 4 | 2104 | 1 | 12 | 1832 |
|  | 10169 | 2 | 0.00% | 0.00% | 0.09% | 0.37% |  | 0 | 0 | 2212 | 2 | 8 | 2138 |
|  | 10168 | 3 | 0.00% | 0.14% | 0.05% | 0.25% |  | 0 | 3 | 2135 | 1 | 5 | 2026 |
|  | *Mean of trials* | | 0.00% | 0.11% | 0.07% | 0.43% | *Totals* | 0 | 7 | 6451 | 4 | 25 | 5996 |
|  | *SD (sample)* | | 0.00% | 0.10% | 0.02% | 0.21% |  |  |  |  |  |  |  |
|  |  |  |  |  |  |  |  |  |  |  |  |  |  |
| ARS317 at CEN5, ∆rad52∆dnl4 | 10238 | 1 | 0.05% | 0.24% | 0.56% | 1.02% |  | 1 | 5 | 2115 | 10 | 18 | 1771 |
|  | 10238 | 2 | 0.00% | 0.33% | 0.64% | 0.85% |  | 0 | 6 | 1838 | 12 | 16 | 1883 |
|  | 10240 | 3 | 0.05% | 0.33% | 0.61% | 1.21% |  | 1 | 7 | 2096 | 14 | 28 | 2312 |
|  | *Mean of trials* | | 0.03% | 0.30% | 0.60% | 1.03% | *Totals* | 2 | 18 | 6049 | 36 | 62 | 5966 |
|  | *SD (sample)* | | 0.03% | 0.05% | 0.04% | 0.18% |  |  |  |  |  |  |  |

S7 Table (continued)

| **Relevant genotype** | **Strain** | **Trial No.** | **Sectoring Frequencies** | | | |  | **Actual Colony Counts** | | | | | |
| --- | --- | --- | --- | --- | --- | --- | --- | --- | --- | --- | --- | --- | --- |
|  |  |  | **Before induction** | | **After Induction** | |  | **Before Induction** | | | **After Induction** | | |
|  |  |  | **Red/White** | **Red/Pink** | **Red/White** | **Red/Pink** |  | **Red/White** | **Red/Pink** | **All colonies** | **Red/White** | **Red/Pink** | **All colonies** |
| No ARS317, ∆rad52∆dnl4 | 10235 | 1 | 0.05% | 0.14% | 0.10% | 0.72% |  | 1 | 3 | 2115 | 2 | 15 | 2080 |
|  | 10235 | 2 | 0.00% | 0.26% | 0.00% | 0.46% |  | 0 | 5 | 1939 | 0 | 10 | 2167 |
|  | 10236 | 3 | 0.00% | 0.26% | 0.14% | 0.72% |  | 0 | 4 | 1526 | 3 | 15 | 2096 |
|  | *Mean of trials* | | 0.02% | 0.22% | 0.08% | 0.63% | *Totals* | 1 | 12 | 5580 | 5 | 40 | 6343 |
|  | *SD (sample)* | | 0.03% | 0.07% | 0.07% | 0.15% |  |  |  |  |  |  |  |
|  |  |  |  |  |  |  |  |  |  |  |  |  |  |
| ARS317 at CEN5 (no arrest) | 9637 | 1 | 0.00% | 0.00% | 0.17% | 1.96% |  | 0 | 0 | 1938 | 6 | 70 | 3563 |
|  | 9637 | 2 | 0.00% | 0.05% | 0.26% | 2.60% |  | 0 | 1 | 2193 | 5 | 50 | 1920 |
|  | 9637 | 3 | 0.00% | 0.00% | 0.35% | 2.86% |  | 0 | 0 | 2076 | 8 | 64 | 2273 |
|  | 9639 | 4 | 0.00% | 0.00% | 0.42% | 2.23% |  | 0 | 0 | 2100 | 10 | 51 | 2381 |
|  | *Mean of trials* | | 0.00% | 0.01% | 0.30% | 2.41% | *Totals* | 0 | 1 | 8307 | 29 | 235 | 10137 |
|  | *SD (sample)* | | 0.00% | 0.03% | 0.11% | 0.40% |  |  |  |  |  |  |  |
|  |  |  |  |  |  |  |  |  |  |  |  |  |  |
| No ARS317 (no arrest) | 9627 | 1 | 0.00% | 0.00% | 0.00% | 0.23% |  | 0 | 0 | 1931 | 0 | 9 | 3887 |
|  | 9627 | 2 | 0.00% | 0.05% | 0.10% | 0.31% |  | 0 | 1 | 1965 | 2 | 6 | 1966 |
|  | 9627 | 3 | 0.00% | 0.00% | 0.00% | 0.13% |  | 0 | 0 | 2178 | 0 | 3 | 2228 |
|  | 9629 | 4 | 0.00% | 0.05% | 0.05% | 0.37% |  | 0 | 1 | 2160 | 1 | 8 | 2156 |
|  | *Mean of trials* | | 0.00% | 0.03% | 0.04% | 0.26% | *Totals* | 0 | 2 | 8234 | 3 | 26 | 10237 |
|  | *SD (sample)* | | 0.00% | 0.03% | 0.05% | 0.10% |  |  |  |  |  |  |  |
