# Supplementary material for: Re-replication of a Centromere Induces Chromosomal Instability and Aneuploidy
Source: PLoS Genet. 2015 Apr 22;11(4):e1005039. doi: 10.1371/journal.pgen.1005039 (PMC4406714; doi:10.1371/journal.pgen.1005039)
Supplement: S8 Table — Mean sectoring frequency for each set of repeated re-replication trials (n ≥ 3) was multiplied by the fraction of sectored colonies that exhibited the distribution of ChrV copy number expected for the relevant segregation event (see Materials and Methods). (DOCX) [file pgen.1005039.s014.docx]

**S8 Table. Calculation of segregation frequencies.** Mean sectoring frequency for each set of repeated re-replication trials (n ≥ 3) was multiplied by the fraction of sectored colonies that exhibited the distribution of Chr5 copy number expected for the relevant segregation event (see Materials and Methods).

| **Red/white sectoring frequency conversion to 2:0 segregation events** | | | |
| --- | --- | --- | --- |
| **Genotype** | **Mean sectoring frequency (see Table S7)** | **Fraction of colonies showing 2:0 distribution of Chr5** | **Estimated frequency of 2:0 segregation events** |
| ARS317 at CEN5 | 0.78% | 9/11 (82%) *see Table S2* | 0.64% |
| No ARS317 | 0.07% | 3/4 (75%) *see Table S2* | 0.05% |
| ARS317 at CEN5 (no arrest) | 0.30% | 8/10 (80%) *see Table S4* | 0.24% |
| No ARS317 (no arrest) | 0.04% | 1/1 (100%) *see Table S4* | 0.04% |
|  |  |  |  |
| **Red/pink sectoring frequency conversion to 2:1 segregation events** | | | |
| **Genotype** | **Mean sectoring frequency (see Table S7)** | **Fraction of colonies showing 2:1 distribution of Chr5** | **Estimated frequency of 2:1 segregation events** |
| ARS317 at CEN5 | 2.42% | 9/11 (82%) *see Table S3* | 1.98% |
| No ARS317 | 0.23% | 4/10 (10%) *see Table S3* | 0.09% |
| ARS317 at CEN5, *rad52∆* | 1.14% | 9/10 (90%) *see Table S3* | 1.03% |
| ARS317 at CEN5, *dnl4∆* | 7.41% | 9/10 (90%) *see Table S3* | 6.67% |
| ARS317 at CEN5, *rad52∆dnl4∆* | 1.03% | 9/10 (90%) *see Table S3* | 0.92% |
| ARS317 at CEN5 (no arrest) | 2.41% | 10/10 (100%) *see Table S5* | 2.41% |
| No ARS317 (no arrest) | 0.26% | 7/10 (70%) *see Table S5* | 0.18% |
