# Supplementary material for: Re-replication of a Centromere Induces Chromosomal Instability and Aneuploidy
Source: PLoS Genet. 2015 Apr 22;11(4):e1005039. doi: 10.1371/journal.pgen.1005039 (PMC4406714; doi:10.1371/journal.pgen.1005039)
Supplement: S9 Table — For each locus, the allele of the MATa parent is listed first. Key features of each strain are in bold. (DOCX) [file pgen.1005039.s015.docx]

**S9 Table. Strains used in this study.** For each locus, the allele of the *MATa* parent is listed first. Key features of each strain are in bold.

| Strain number | Genotype |
| --- | --- |
| YJL8590 | *ORC2-(NotI, SgrAI)/ORC2-(NotI, SgrAI) orc6(S116A)/orc6(S116A) leu2/leu2 ura3::{ACT1term-pGAL1/10-CDC6term}/ura3::{ACT1term-pGAL1/10-CDC6term} trp1-289/trp1-289 ade2/ade2 ade3/ade3 MCM7-2NLS/MCM7-2NLS bar1::LEU2/bar1::LEU2 HMRa::HPHMX/HMRa::HPHMX* ***ChrXVI_550kb::{ade3-2p, ARS317, kanMX}/ChromXVI*** *MATa/MATα* |
| YJL9627 | *ORC2-(NotI, SgrAI)/ORC2-(NotI, SgrAI) orc6(S116A)/orc6(S116A) leu2/leu2 ura3::{ACT1term-pGAL1/10-delntCDC6,cdk2A-CDC6term}/ura3::{ACT1term-pGAL1/10-delntCDC6,cdk2A-CDC6term} trp1-289/trp1-289 ade2/ade2 ade3/ade3 MCM7-2NLS/MCM7-2NLS bar1/bar1 HMRa::HPHMX/HMRa::HPHMX* ***ChrV_160::{KanMX, ade3-2p}/ChrV_160::URA3 ChrV_548::{NatMX, ade3-2w}/ChrV_548*** *MATa/MATα* |
| YJL9629 | *ORC2-(NotI, SgrAI)/ORC2-(NotI, SgrAI) orc6(S116A)/orc6(S116A) leu2/leu2 ura3::{ACT1term-pGAL1/10-delntCDC6,cdk2A-CDC6term}/ura3::{ACT1term-pGAL1/10-delntCDC6,cdk2A-CDC6term} trp1-289/trp1-289 ade2/ade2 ade3/ade3 MCM7-2NLS/MCM7-2NLS bar1/bar1 HMRa::HPHMX/HMRa::HPHMX* ***ChrV_160::{KanMX, ade3-2p}/ChrV_160::URA3 ChrV_548::{NatMX, ade3-2w}/ChrV_548*** *MATa/MATα* |
| YJL9631 | *ORC2-(NotI, SgrAI)/ORC2-(NotI, SgrAI) orc6(S116A)/orc6(S116A) leu2/leu2 ura3::{ACT1term-pGAL1/10-delntCDC6,cdk2A-CDC6term}/ura3::{ACT1term-pGAL1/10-delntCDC6,cdk2A-CDC6term} trp1-289/trp1-289 ade2/ade2 ade3/ade3 MCM7-2NLS/MCM7-2NLS bar1/bar1 HMRa::HPHMX/HMRa::HPHMX* ***ChrV_160::{KanMX, ade3-2p}/ChrV_160::URA3 ChrV_548::{NatMX, ade3-2w, ARS317}/ChrV_548*** *MATa/MATα* |
| YJL9633 | *ORC2-(NotI, SgrAI)/ORC2-(NotI, SgrAI) orc6(S116A)/orc6(S116A) leu2/leu2 ura3::{ACT1term-pGAL1/10-delntCDC6,cdk2A-CDC6term}/ura3::{ACT1term-pGAL1/10-delntCDC6,cdk2A-CDC6term} trp1-289/trp1-289 ade2/ade2 ade3/ade3 MCM7-2NLS/MCM7-2NLS bar1/bar1 HMRa::HPHMX/HMRa::HPHMX* ***ChrV_160::{KanMX, ade3-2p}/ChrV_160::URA3 ChrV_548::{NatMX, ade3-2w, ARS317}/ChrV_548*** *MATa/MATα* |
| YJL9637 | *ORC2-(NotI, SgrAI)/ORC2-(NotI, SgrAI) orc6(S116A)/orc6(S116A) leu2/leu2 ura3::{ACT1term-pGAL1/10-delntCDC6,cdk2A-CDC6term}/ura3::{ACT1term-pGAL1/10-delntCDC6,cdk2A-CDC6term} trp1-289/trp1-289 ade2/ade2 ade3/ade3 MCM7-2NLS/MCM7-2NLS bar1/bar1 HMRa::HPHMX/HMRa::HPHMX* ***ChrV_160::{KanMX, ade3-2p, ARS317}/ChrV_160::URA3 ChrV_548::{NatMX, ade3-2w}/ChrV_548*** *MATa/MATα* |
| YJL9639 | *ORC2-(NotI, SgrAI)/ORC2-(NotI, SgrAI) orc6(S116A)/orc6(S116A) leu2/leu2 ura3::{ACT1term-pGAL1/10-delntCDC6,cdk2A-CDC6term}/ura3::{ACT1term-pGAL1/10-delntCDC6,cdk2A-CDC6term} trp1-289/trp1-289 ade2/ade2 ade3/ade3 MCM7-2NLS/MCM7-2NLS bar1/bar1 HMRa::HPHMX/HMRa::HPHMX* ***ChrV_160::{KanMX, ade3-2p, ARS317}/ChrV_160::URA3 ChrV_548::{NatMX, ade3-2w}/ChrV_548*** *MATa/MATα* |
| YJL10164 | *ORC2-(NotI, SgrAI)/ORC2-(NotI, SgrAI) orc6(S116A)/orc6(S116A) leu2/leu2 ura3::{ACT1term-pGAL1/10-delntCDC6,cdk2A-CDC6term}/ura3::{ACT1term-pGAL1/10-delntCDC6,cdk2A-CDC6term} trp1-289/trp1-289 ade2/ade2 ade3/ade3 MCM7-2NLS/MCM7-2NLS bar1/bar1 HMRa::HPHMX/HMRa::HPHMX* ***ChrV_160::{KanMX, ade3-2p}/ChrV_160::URA3 ChrV_548::{NatMX, ade3-2w}/ChrV_548 ∆rad52::LEU2/∆rad52::LEU2*** *MATa/MATα* |
| YJL10165 | *ORC2-(NotI, SgrAI)/ORC2-(NotI, SgrAI) orc6(S116A)/orc6(S116A) leu2/leu2 ura3::{ACT1term-pGAL1/10-delntCDC6,cdk2A-CDC6term}/ura3::{ACT1term-pGAL1/10-delntCDC6,cdk2A-CDC6term} trp1-289/trp1-289 ade2/ade2 ade3/ade3 MCM7-2NLS/MCM7-2NLS bar1/bar1 HMRa::HPHMX/HMRa::HPHMX* ***ChrV_160::{KanMX, ade3-2p}/ChrV_160::URA3 ChrV_548::{NatMX, ade3-2w}/ChrV_548 ∆rad52::LEU2/∆rad52::LEU2*** *MATa/MATα* |

**S9 Table (continued)**

| Strain number | Genotype |
| --- | --- |
| YJL10168 | *ORC2-(NotI, SgrAI)/ORC2-(NotI, SgrAI) orc6(S116A)/orc6(S116A) leu2/leu2 ura3::{ACT1term-pGAL1/10-delntCDC6,cdk2A-CDC6term}/ura3::{ACT1term-pGAL1/10-delntCDC6,cdk2A-CDC6term} trp1-289/trp1-289 ade2/ade2 ade3/ade3 MCM7-2NLS/MCM7-2NLS bar1/bar1 HMRa::HPHMX/HMRa::HPHMX* ***ChrV_160::{KanMX, ade3-2p}/ChrV_160::URA3 ChrV_548::{NatMX, ade3-2w}/ChrV_548 ∆dnl4::LEU2/∆dnl4::LEU2*** *MATa/MATα* |
| YJL10169 | *ORC2-(NotI, SgrAI)/ORC2-(NotI, SgrAI) orc6(S116A)/orc6(S116A) leu2/leu2 ura3::{ACT1term-pGAL1/10-delntCDC6,cdk2A-CDC6term}/ura3::{ACT1term-pGAL1/10-delntCDC6,cdk2A-CDC6term} trp1-289/trp1-289 ade2/ade2 ade3/ade3 MCM7-2NLS/MCM7-2NLS bar1/bar1 HMRa::HPHMX/HMRa::HPHMX* ***ChrV_160::{KanMX, ade3-2p}/ChrV_160::URA3 ChrV_548::{NatMX, ade3-2w}/ChrV_548 ∆dnl4::LEU2/∆dnl4::LEU2*** *MATa/MATα* |
| YJL10171 | *ORC2-(NotI, SgrAI)/ORC2-(NotI, SgrAI) orc6(S116A)/orc6(S116A) leu2/leu2 ura3::{ACT1term-pGAL1/10-delntCDC6,cdk2A-CDC6term}/ura3::{ACT1term-pGAL1/10-delntCDC6,cdk2A-CDC6term} trp1-289/trp1-289 ade2/ade2 ade3/ade3 MCM7-2NLS/MCM7-2NLS bar1/bar1 HMRa::HPHMX/HMRa::HPHMX* ***ChrV_160::{KanMX, ade3-2p, ARS317}/ChrV_160::URA3 ChrV_548::{NatMX, ade3-2w}/ChrV_548 ∆rad52::LEU2/∆rad52::LEU****2 MATa/MATα* |
| YJL10172 | *ORC2-(NotI, SgrAI)/ORC2-(NotI, SgrAI) orc6(S116A)/orc6(S116A) leu2/leu2 ura3::{ACT1term-pGAL1/10-delntCDC6,cdk2A-CDC6term}/ura3::{ACT1term-pGAL1/10-delntCDC6,cdk2A-CDC6term} trp1-289/trp1-289 ade2/ade2 ade3/ade3 MCM7-2NLS/MCM7-2NLS bar1/bar1 HMRa::HPHMX/HMRa::HPHMX* ***ChrV_160::{KanMX, ade3-2p, ARS317}/ChrV_160::URA3 ChrV_548::{NatMX, ade3-2w}/ChrV_548 ∆rad52::LEU2/∆rad52::LEU2*** *MATa/MATα* |
| YJL10176 | *ORC2-(NotI, SgrAI)/ORC2-(NotI, SgrAI) orc6(S116A)/orc6(S116A) leu2/leu2 ura3::{ACT1term-pGAL1/10-delntCDC6,cdk2A-CDC6term}/ura3::{ACT1term-pGAL1/10-delntCDC6,cdk2A-CDC6term} trp1-289/trp1-289 ade2/ade2 ade3/ade3 MCM7-2NLS/MCM7-2NLS bar1/bar1 HMRa::HPHMX/HMRa::HPHMX* ***ChrV_160::{KanMX, ade3-2p, ARS317}/ChrV_160::URA3 ChrV_548::{NatMX, ade3-2w}/ChrV_548 ∆dnl4::LEU2/∆dnl4::LEU****2 MATa/MATα* |
| YJL10177 | *ORC2-(NotI, SgrAI)/ORC2-(NotI, SgrAI) orc6(S116A)/orc6(S116A) leu2/leu2 ura3::{ACT1term-pGAL1/10-delntCDC6,cdk2A-CDC6term}/ura3::{ACT1term-pGAL1/10-delntCDC6,cdk2A-CDC6term} trp1-289/trp1-289 ade2/ade2 ade3/ade3 MCM7-2NLS/MCM7-2NLS bar1/bar1 HMRa::HPHMX/HMRa::HPHMX* ***ChrV_160::{KanMX, ade3-2p, ARS317}/ChrV_160::URA3 ChrV_548::{NatMX, ade3-2w}/ChrV_548 ∆dnl4::LEU2/∆dnl4::LEU2*** *MATa/MATα* |
| YJL10235 | *ORC2-(NotI, SgrAI)/ORC2-(NotI, SgrAI) orc6(S116A)/orc6(S116A) leu2/leu2 ura3::{ACT1term-pGAL1/10-delntCDC6,cdk2A-CDC6term}/ura3::{ACT1term-pGAL1/10-delntCDC6,cdk2A-CDC6term} trp1-289/trp1-289 ade2/ade2 ade3/ade3 MCM7-2NLS/MCM7-2NLS bar1/bar1 HMRa::HPHMX/HMRa::HPHMX* ***ChrV_160::{KanMX, ade3-2p}/ChrV_160::URA3 ChrV_548::{NatMX, ade3-2w}/ChrV_548 ∆dnl4::LEU2/∆dnl4::LEU2 ∆rad52::URA3/∆rad52::NatMX*** *MATa/MATα* |
| YJL10236 | *ORC2-(NotI, SgrAI)/ORC2-(NotI, SgrAI) orc6(S116A)/orc6(S116A) leu2/leu2 ura3::{ACT1term-pGAL1/10-delntCDC6,cdk2A-CDC6term}/ura3::{ACT1term-pGAL1/10-delntCDC6,cdk2A-CDC6term} trp1-289/trp1-289 ade2/ade2 ade3/ade3 MCM7-2NLS/MCM7-2NLS bar1/bar1 HMRa::HPHMX/HMRa::HPHMX* ***ChrV_160::{KanMX, ade3-2p}/ChrV_160::URA3 ChrV_548::{NatMX, ade3-2w}/ChrV_548 ∆dnl4::LEU2/∆dnl4::LEU2 ∆rad52::URA3/∆rad52::NatMX*** *MATa/MATα* |
| YJL10238 | *ORC2-(NotI, SgrAI)/ORC2-(NotI, SgrAI) orc6(S116A)/orc6(S116A) leu2/leu2 ura3::{ACT1term-pGAL1/10-delntCDC6,cdk2A-CDC6term}/ura3::{ACT1term-pGAL1/10-delntCDC6,cdk2A-CDC6term} trp1-289/trp1-289 ade2/ade2 ade3/ade3 MCM7-2NLS/MCM7-2NLS bar1/bar1 HMRa::HPHMX/HMRa::HPHMX* ***ChrV_160::{KanMX, ade3-2p, ARS317}/ChrV_160::URA3 ChrV_548::{NatMX, ade3-2w}/ChrV_548 ∆dnl4::LEU2/∆dnl4::LEU2 ∆rad52::URA3/∆rad52::NatMX*** *MATa/MATα* |

**S9 Table (continued)**

| Strain number | Genotype |
| --- | --- |
| YJL10240 | *ORC2-(NotI, SgrAI)/ORC2-(NotI, SgrAI) orc6(S116A)/orc6(S116A) leu2/leu2 ura3::{ACT1term-pGAL1/10-delntCDC6,cdk2A-CDC6term}/ura3::{ACT1term-pGAL1/10-delntCDC6,cdk2A-CDC6term} trp1-289/trp1-289 ade2/ade2 ade3/ade3 MCM7-2NLS/MCM7-2NLS bar1/bar1 HMRa::HPHMX/HMRa::HPHMX* ***ChrV_160::{KanMX, ade3-2p, ARS317}/ChrV_160::URA3 ChrV_548::{NatMX, ade3-2w}/ChrV_548 ∆dnl4::LEU2/∆dnl4::LEU2 ∆rad52::URA3/∆rad52::NatMX*** *MATa/MATα* |
| YJL10665 | *ORC2-(NotI, SgrAI) orc6(S116A) leu2 ura3::{ACT1term-pGAL1/10-delntCDC6,cdk2A-CDC6term} trp1-289::{GFP-TUB1, TRP1} ade2::{pCUP1-tetR-tdTomato, URA3, ADE2} ade3 MCM7-2NLS bar1 his7? sap3? HMRa::HPHMX* ***ChrV_160::{KanMX, ade3-2p} ChrV_548::{NatMX, ade3-2w}*** ***Chr5_151::{LEU2, tetOx128}*** *MATa* |
| YJL10666 | *ORC2-(NotI, SgrAI) orc6(S116A) leu2 ura3::{ACT1term-pGAL1/10-delntCDC6,cdk2A-CDC6term} trp1-289::{GFP-TUB1, TRP1} ade2::{pCUP1-tetR-tdTomato, URA3, ADE2} ade3 MCM7-2NLS bar1 his7? sap3? HMRa::HPHMX* ***ChrV_160::{KanMX, ade3-2p} ChrV_548::{NatMX, ade3-2w}*** ***Chr5_151::{LEU2, tetOx128}*** *MATa* |
| YJL10671 | *ORC2-(NotI, SgrAI) orc6(S116A) leu2 ura3::{ACT1term-pGAL1/10-delntCDC6,cdk2A-CDC6term} trp1-289::{GFP-TUB1, TRP1} ade2::{pCUP1-tetR-tdTomato, URA3, ADE2} ade3 MCM7-2NLS bar1 his7? sap3? HMRa::HPHMX* ***ChrV_160::{KanMX, ade3-2p, ARS317} ChrV_548::{NatMX, ade3-2w}*** ***Chr5_151::{LEU2, tetOx128}*** *MATa* |
| YJL10672 | *ORC2-(NotI, SgrAI) orc6(S116A) leu2 ura3::{ACT1term-pGAL1/10-delntCDC6,cdk2A-CDC6term} trp1-289::{GFP-TUB1, TRP1} ade2::{pCUP1-tetR-tdTomato, URA3, ADE2} ade3 MCM7-2NLS bar1 his7? sap3? HMRa::HPHMX* ***ChrV_160::{KanMX, ade3-2p, ARS317} ChrV_548::{NatMX, ade3-2w}*** ***Chr5_151::{LEU2, tetOx128}*** *MATa* |
