# Supplementary material for: Re-replication of a Centromere Induces Chromosomal Instability and Aneuploidy
Source: PLoS Genet. 2015 Apr 22;11(4):e1005039. doi: 10.1371/journal.pgen.1005039 (PMC4406714; doi:10.1371/journal.pgen.1005039)
Supplement: S10 Table — Cassettes and fragments were targeted for homologous recombination into the genome (see Materials and Methods) by flanking sequences mapping to the left and right as determined by the conventional left to right polarity of each chromosome. Sequence is reported in the 5’ to 3’ direction of the Watson strand of the S288C reference sequence from the Saccharomyces Genome Database (version R64–1–1). (DOCX) [file pgen.1005039.s016.docx]

S10 Table. Flanking homologies for integration of re-replication and gene deletion fragments. Cassettes and fragments were targeted for homologous

recombination into the genome (see Materials and Methods) by flanking sequences mapping to the left and right as determined by the conventional left to right

polarity of each chromosome. Sequence is reported in the 5’ to 3’ direction of the Watson strand of the S288C reference sequence from the Saccharomyces

Genome Database (version R64-1-1).

| **Homology for ChrV_160** | |
| --- | --- |
| *Left homology* | *Right homology* |
| AGATTCCAGAGGTTGCTAGGCTATCCAGTTTATGGCGATGAGGTAGGAAAAGTTGGTGTTCATTTGGCGTTGAATACTTCTGGGAAAGACAAGGTTCAGTTTGTCTCCAGCAAGGACATTCTTGACATCTCAGCTTCTCTGGAGAAGATTGCTACCTAAA | CAACCAACGGCTTCTTCTCGCAGCTACAGTTGTATTTTACTGTGACGTACTTTTGCGCGCGTACGGGTTCAATGAATTACGGATTTTCAACAACTTCACACAATCATAGCTTTTTTTACTTTTTCGCCTCATTATTTTCTTTAAAGAAACGATGATAAACAGAGATTTATCGTCATGTTATTTCGTTCGTATATATCGTATTTGCGTTTTAGCTGTATAAAGTAAAATACTATATCTAACGTGGCTTTTTTTATTTCCCCGTCTGCCCTCTTATGCTCATAGAAAACACTAATGATCGGTTTGGTATCGTCATAGATGCAGGGTCTTCGGGTTCGAGAATCCATGTGTTTAAGTGGCAGGACACGGAATCGCTTCTTCATGCAACAAACCAGGACTCACAGTCCATATTACAATCAGTACCTCATATTCATCAAGAAAAAGACTGGACTTTTAAGCTGAATCCAGGCTTGTCGAGTTTTGAAAAAAAACCTCAAGATGCGTACAAATCTC |
| **Homology for ChrV_548** | |
| *Left homology* | *Right homology* |
| GCCATTCTATGTCTGATCCCGGTACTACCTACAGAACTAGAGACGAGATTCAGCATATGAGATCCAAGAACGATCCAATTGCTGGTCTTAAGATGCATTTGATTGATCTAGGTATTGCCACTGAAGCTGAAGTCAAAGCTTACGACAAGTCCGCTAGAAAATACGTTGACGAACAAGTTGAATTAGCTGATGCTGCTCCTCCTCCAGAAGCCAAATTATCCATCTTGTTTGAAGACGTCTACGTGAAAGGTACAGAAACTCCAACCCTAAGAGGTAGGATCCCTGAAGATACTTGGGACTTCAAAAAGCAAGGTTTTGCCTCTAGGGATTAATTAAATCGTAAGGAAAAATAAAATAATAGTGCTGTGATCGCATGATATTCTTCC | CTGGAAGCGCCATTTTATAGCAAGAAATGTAAGTCAAGTATATTTTAACTGTATATCAACAATATAGCTCTTTTTTATGCCTTGTTGTTTTTCTTCGGGTTTTCCCCACACATTGTGTGGAGAGATAGTTATTAACAGACCGGAAAATAGCCGCCCAAGGATAAACTTTTATATAAAGGGAAGGGTAGTTGACCCAAAAAATTTGGATTCTACTTTTCCAGATTTACTTTCACCCTTTTATATTTGCTGTAGTCTGTTATGCCAATCAGGAAAGCATTTGAACAAATATGTCTGTTACAGGAACTGAGATCGATAGTGATACAGCAAAAAATATTCTTAGTGTAGATGAATTACAGAACTATGGTATTAATGCCTCAGATCTTCAAAAATTGAAGTCTGGTGGGATATACACAGTCAATGTATGTTATAATAACATTTTTAAAACCTCTGCTGTAGAGGTTCTTTCCCCCTTTCTTTTACTAACTAATAATTTGGAAAGGAACTTTTATAGACCGTTTTGTCAACAACAAGAAGACATCTATGTAAAATTAAAGGGTTAAGTGAGGTGAAAGT |

| **Homology for *rad52∆*** | |
| --- | --- |
| *Left homology* | *Right homology* |
| AACTAGAGGATTTTGGAGTAATAAATAATGATGCAAATTTTTTATTTGTTTCGGCCAGGAAGCGTT | CCAACAACACACCAAAGCCACCAGAACCTTCAGCAGTTCTTGGCAACCTCCTTGTTTGCAT |
| **Homology for *dnl4∆*** | |
| *Left homology* | *Right homology* |
| TACATATGTAGGATAGTATTAAATAAACTTCAAAAAATTAAGCCTCCGCAAAACGCACCA | ACCTAAATAATCCGTTACTATTTCCTTCAGTTCTAGATTTTTATTTTAGTATTTATTTTCCAC |
| **Homology for *HMRa∆*** | |
| *Left homology* | *Right homology* |
| TTGTTTACTTTTTCTATCAGTGTTTTCAATTTTTTATTAAACAATGTTTGATTTTTTAAATCGCAATTTAATACC | TTAATACCTTTAAATGTTGAGGTAAATAGCTATTTTCTCTCTTCTTTTCCTTTAGTTGGAATTTGCACAAGAAAA |
| **Homology for *TRP1* insertion at ChrV_151 (target for pSR14 integration)** | |
| *Left homology* | *Right homology* |
| TCTTCATTAACAGGGGAACGCTTGCCTACCATCAAGCCCATTCAATGCAGATGTGATTAA | TTGGTAAACAAAGGGCCAAGCAAAAATACATCTCTCCTACATGCTACATAAGTCCGAGA |
