# Supplementary material for: Re-replication of a Centromere Induces Chromosomal Instability and Aneuploidy
Source: PLoS Genet. 2015 Apr 22;11(4):e1005039. doi: 10.1371/journal.pgen.1005039 (PMC4406714; doi:10.1371/journal.pgen.1005039)
Supplement: S11 Table — Uppercase indicates where oligo hybridizes to the PCR template; lowercase corresponds to chromosomal sequences used to target integration of fragment (see Materials and Methods). (DOCX) [file pgen.1005039.s017.docx]

S11 Table. Oligonuclotides used to PCR fragments for strain construction. Uppercase indicates where oligo hybridizes to the PCR template; lowercase corresponds to chromosomal sequences used to target integration of fragment (see Materials and Methods).

| **Oligo number** | **Sequence (*5’ to 3’*)** | **Disruption** |
| --- | --- | --- |
| OJL1639 | attaaacaatgtttgattttttaaatcgcaatttaataccCGGATCCCCGGGTTAATTAA | *HMRa∆* |
| OJL1642 | agagaaaatagctatttacctcaacatttaaaggtattaaCATCGATGAATTCGAGCTCG | *HMRa∆* |
| OJL2200 | ttgtttactttttctatcagtgttttcaattttttattaaacaatgtttgattttttaaa | *HMRa∆* |
| OJL2201 | ttttcttgtgcaaattccaactaaaggaaaagaagagagaaaatagctatttacctcaac | *HMRa∆* |
| OJL2821 | gcttctctggagaagattgctacctaaaGAGCAGATTGTACTGAGAGTG | *URA3* at *CEN5* |
| OJL2822 | ctgtagctgcgagaagaagccgttggttgaGCATCTGTGCGGTATTTCAC | *URA3* at *CEN5* |
| OJL2823 | tctccagcaaggacattcttgacatctcagcttctctggagaagattgct | *URA3* at *CEN5* |
| OJL2824 | cgcgcaaaagtacgtcacagtaaaatacaactgtagctgcgagaagaagc | *URA3* at *CEN5* |
| OJL2117 | gaaggttctggtggctttggtgtgttgttgGAGCAGATTGTACTGAGAGTG | *rad52∆* (with *LEU2* or *URA3*) |
| OJL2118 | aatgatgcaaattttttatttgtttcggccaggaagcgttGCATCTGTGCGGTATTTCAC | *rad52∆* (with *LEU2* or *URA3*) |
| OJL1753 | aagaactgctgaaggttctggtggctttggtgtgttgttgCGGATCCCCGGGTTAATTAA | *rad52∆* (with *NatMX*) |
| OJL1754 | aatgatgcaaattttttatttgtttcggccaggaagcgttCATCGATGAATTCGAGCTCG | *rad52∆* (with *NatMX*) |
| OJL2119 | atgcaaacaaggaggttgccaagaactgctgaaggttctggtggctttgg | *rad52∆* |
| OJL2120 | aactagaggattttggagtaataaataatgatgcaaattttttatttgtttcggc | *rad52∆* |
| OJL2097 | gaactgaaggaaatagtaacggattatttaggtGAGCAGATTGTACTGAGAGTG | *dnl4∆* |
| OJL2098 | caaaaaattaagcctccgcaaaacgcaccaGCATCTGTGCGGTATTTCAC | *dnl4∆* |
| OJL2099 | gtggaaaataaatactaaaataaaaatctagaactgaaggaaatagtaacgg | *dnl4∆* |
| OJL2100 | tacatatgtaggatagtattaaataaacttcaaaaaattaagcctccgc | *dnl4∆* |
| OJL3113 | atcaagcccattcaatgcagatgtgattaaAGCGGATGCCGGGAGCAGAC | *TRP1* amplification for pSR14 targeting |
| OJL3114 | tgtatttttgcttggccctttgtttaccaaGTGAGCTGATACCGCTCGCC | *TRP1* amplification for pSR14 targeting |
| OJL3115 | tcttcattaacaggggaacgcttgcctaccATCAAGCCCATTCAATGCAG | *TRP1* amplification for pSR14 targeting |
| OJL3116 | tctcggacttatgtagcatgtaggagagaTGTATTTTTGCTTGGCCCTTT | *TRP1* amplification for pSR14 targeting |
